# MiR-641 targets TMEFF2/MEK/PI3K to promote stem cell characteristics of pancreatic cancer cells

**DOI:** 10.1007/s12672-026-04584-2

**Published:** 2026-02-07

**Authors:** Hongchao Han, Aikun Wang

**Affiliations:** Department of General Surgery, Affiliated Hospital 6 of Nantong University, Yanchen, 224000 Jiangsu PR China

**Keywords:** MiR-641, TMEFF2, Stem cell characteristics, Pancreatic cancer

## Abstract

**Background:**

Pancreatic cancer is a common malignant tumor. We focused on exploring the function of miR-641 in stem cell characteristics for pancreatic cancer cells.

**Method:**

MiR-641 expression was analyzed based on The Cancer Genome Atlas (TCGA) pancreatic cancer database and clinical samples. The miR-641 knockdown within pancreatic cancer was performed by cell transfection. CCK8, transwell, and flow cytometry were performed for analyzing cell growth, invasion as well as stem-cell-like features separately. Meanwhile, this study carried out the dual luciferase reporter gene assay. *In viv*o xenograft tumor assay was also performed.

**Results:**

MiR-641 expression increased in clinical pancreatic cancer tissues and cells compared with normal cells. MiR-641 predicted poor survival rate of pancreatic cancer patients. MiR-641 down-regulation inhibited pancreatic cancer cell proliferation, clone forming ability, invasion and migration. Down-regulation of miR-641 inhibited the capability of sphere-forming, reduced CD133 + and CD44 + cell quantities, and suppressed CD133, CD44, and Oct4 expression in Down-regulation of miR-641 cells. MiR-641 targeted TMEFF2/MEK/PI3K for promoting pancreatic cancer cells’ stem-cell-like characteristics. MiR-641 also promoted tumor growth in vivo.

**Conclusion:**

MiR-641 acts as an oncogene that promotes pancreatic cancer cell growth, invasion as well as stem-cell-like features, which is realized by regulating the expression of TMEFF2.

**Supplementary Information:**

The online version contains supplementary material available at 10.1007/s12672-026-04584-2.

## Introduction

Pancreatic cancer accounts for a frequently seen digestive tract cancer, which can hardly be diagnosed and treated because of its insidious onset and rapid metastasis [1]. It represents a cancer with poorest prognostic outcome, and the 5-year survival rate is approximately 10%. Pancreatic cancer shows markedly increasing incidence rate and mortality recently, with a higher male to female ratio [2]. Around 90% of pancreatic cancer cases are glandular epithelium-derived ductal adenocarcinomas. The early clinical manifestations of pancreatic cancer are often nonspecific and easy to be ignored, leading to difficulties in early diagnosis. Prominent symptoms include unexplained anorexia, indigestion, weight loss, irregular bowel movements, especially an increase in bowel movements after consuming greasy foods [3]. In addition, abdominal discomfort or pain is also a common symptom, with about half of patients experiencing abdominal pain as the first symptom, and about 20% of patients experiencing abdominal pain radiating to the back, left shoulder, etc., often related to body position. The cause of pancreatic cancer is not completely clear, but some identified risk factors include long-time heavy smoking, alcohol consumption (like beer), refined flour food, high-fat and high-animal-protein diet, cholelithiasis, diabetes, and chronic pancreatitis [4]. In addition, gastrectomy is a risk factor related to pancreatic cancer. Pancreatic cancer can be treated through surgical resection, chemotherapy or radiotherapy, but with poor treatment effect, low cure rate, and high operative mortality. In general, pancreatic cancer is a very dangerous malignant tumor, which needs early diagnosis and treatment. Tumor stem cells account for a low fraction in tumors, with self-renewal and multi-lineage differentiation capacities, and are considered the root cause of tumor occurrence, recurrence, and metastasis. Pancreatic cancer shows a high malignancy grade, whose treatment and prognosis are relatively difficult [5]. As found in recent research, tumor stem cells play a key role in pancreatic cancer genesis, progression, treatment and prognosis [6]. On the one hand, tumor stem cells promote pancreatic cancer cell growth, invasion and migration through activating multiple pathways, including Wnt/β-Catenin, Notch, Hedgehog, and PI3K/Akt [7]. On the other hand, pancreatic cancer cells can form tumor stem cells in hypoxic and nutrient deficient microenvironments, and the presence of tumor stem cells can also affect the metabolic mode of pancreatic cancer cells. In addition, researchers have also discovered some surface markers related to pancreatic cancer stem cells, like CD24, CD44, ESA, etc [8]. These biomarkers are adopted for isolating pancreatic cancer stem cells and detecting them within clinical samples. The treatment strategies for tumor stem cells are also being studied. Certain therapeutic agents are suggested to suppress tumor stem cell activity or induce their differentiation, thereby reducing the malignancy of pancreatic cancer. In addition, immunotherapy is also a promising method for treating pancreatic cancer through promoting patient’s immune capacity against tumor stem cells [9]. In summary, tumor stem cells are an important topic in pancreatic cancer studies, while further research on them will contribute to understanding the pancreatic cancer occurrence and development mechanisms, and provide new ideas for treatment and prevention.

MicroRNAs (miRNAs) are non-coding single-stranded RNA molecules approximately 22 nucleotides long. These molecules can be found from various organisms, from viruses to humans. MiRNAs are important for regulating gene expression post-transcriptionally in both animals and plants [10]. They can bind to mRNA and block the protein-coding gene translation, preventing protein production. Every miRNA may possess several target genes, whereas multiple miRNAs may modulate one gene. The complicated regulatory network allows miRNAs to control several gene expression via individual miRNAs or through combining multiple miRNAs to fine-tune the expression of specific genes. miRNAs may modulate approximately 1/3 of human genes. miRNA biogenesis can be relatively complicated, which typically includes several steps. First, the precursor molecule of miRNA (pri-miRNA) is transcribed by a specific enzyme system within the cell. Then, the pri-miRNA undergoes a series of cleavage and processing reactions to form mature miRNA. At last, this formed mature miRNA can combine with RNA-induced silencing complex (RISC) and interact with target mRNAs. miRNAs are important for various biological events, like cell differentiation, cell cycle control, immune response, and development regulation. For example, miR-21, the recognized oncogenic miRNA, participates in almost all cancers [11]. Other miRNAs regulate embryonic and infant development, including nearly all organ development including bone, muscle, heart and brain. miRNAs are non-coding RNA molecules that can post-transcriptionally regulate cells and modulate several target gene expression. Research has shown that MicroRNAs are related to tumor stem cell self-renewal and differentiation, which have an impact on their characteristics. MiRNAs can promote tumor stem cell self-renewal and differentiation. For instance, miR-302 promotes ovarian cancer stem cell self-renewal, while miR-138 can enhance breast cancer stem cell differentiation [12]. MiRNAs can inhibit tumor stem cell proliferation and self-renewal. For example, miR-145 can inhibit colon cancer stem cell self-renewal, while miR-21 can suppress pancreatic cancer stem cell growth. Additionally, miR can also affect cancer stem cell migration and invasion. For instance, miR-21 can accelerate cancer stem cell invasion and migration, while miR-34a has an opposite effect. Effects of miRs on cancer stem cell characteristics are multifaceted, including self-renewal, differentiation, proliferation, invasion, and metastasis [13]. Therefore, regulating miRs may become a new approach for treating cancer. MiR-641 is expressed differently in diverse cancers.

MiR-641 shows low expression within gliomas, lung cancers, and gastric cancers, playing a role of the tumor suppressor through suppressing tumor cells’ malignant phenotypes [14, 15]. On the other hand, miR-641 is up-regulated in pancreatic cancer and cervical cancer [16, 17]. Previously, we reported that miR-641 showed over-expression within pancreatic cancer, which could inhibit cell growth and invasion of pancreatic cancer [16]. This work analyzed the functions of miR-641 in pancreatic cancer development and progression as well as in tumor stem cell characteristics and its mechanism of action.

## Materials and methods

### Clinical sample collection

There were altogether 60 pancreatic cancer cases who received surgical treatment at the Affiliated Hospital 6 of Nantong University from October 2017 to September 2020 included in this study. They provided written informed consents for participation. The ethics committee of Affiliated Hospital 6 of Nantong University approved this work, and it was conducted following the Declaration of Helsinki (No. YCN-0020–14 J).

### Cell lines and treatment

In the present work, we obtained pancreatic cancer cells (SW1990, CFPAC-1, BxPC-3 and PANC-1) and normal human pancreatic ductal epithelial cells (HPDE6-C7) in American Type Culture Collection (ATCC, Manassas, VA, USA) and fostered them individually within Dulbecco’s modified Eagle’s medium (DMEM, Zeye Biotechnology, Shanghai, China) that contained 10% fetal bovine serum (FBS, Beyotime, Shanghai, China) under 37 °C with 5% CO_2_.

For regulating miR-641 expression, pancreatic cancer cells underwent separate transfection by miR-641 inhibitors and scramble (GeneChem, Shanghai, China). Briefly, pancreatic cancer cells reaching 85% confluency were collected and prepared into serum-free DMEM cell suspension (1 × 10^6^ cells/mL). Thereafter, cell suspension (1 mL) was harvested to foster in 6-well plates. Then Lipofectamine 3000 (Thermo Fisher Scientific, Shanghai, China) was adopted for transfection according to the product specification. Thereafter, DMEM containing 10% FBS was added to further foster cells under 37 °C with 5% CO_2_ for a 48-h period. qRT-PCR was performed to analyze transfection efficiency. ‌PANC-1 cells were transfected with 2 µg TMEFF2 overexpression plasmid using Lipofectamine 3000 for 48 h. Subsequently, cells received either: ‌(a) 2 µg MEK overexpression plasmid (24 h transfection), or ‌(b) 20 µM PI3K agonist 740Y-P (24 h treatment). Western blot and functional assays were performed 72 h post-initial transfection.

### CCK8 assay

Cells were harvested, dispersed in DMEM supplemented with 10% FBS, and seeded into 96-well plates at a density of 1 × 10⁵ cells/mL (100 µL per well). After allowing cells to adhere overnight (or for X hours) in a humidified incubator at 37 °C with 5% CO₂, the culture medium was replaced with fresh medium. CCK-8 reagent (10 µL per well) was then added directly to the culture medium. The plates were incubated for an additional 1–4 h (optimize time based on your cell type and protocol) under the same conditions. The absorbance at 450 nm was immediately measured using a microplate reader (Molecular Devices, Shanghai, China).

### Cell colony formation assay

Pancreatic cancer cells were incubated for 24 h, then collected and resuspended in DMEM with 10% FBS. The single cell suspension (2 mL containing 800 cells) was seeded into sterile 60 mm dishes and cultured for 10 days under standard conditions (37 °C, 5% CO₂) with medium replacement every two days. After 10 days, colonies were fixed with 4% paraformaldehyde for 10 min and stained with 0.1% crystal violet for an additional 10 min. Finally, colonies containing more than 50 cells were counted under a microscope.

### Sphere culture

The logarithmic-phase cells were trypsinized prior to 3 min of centrifugation at 1000 r/min. Later, cells on the centrifuge tube bottom were rinsed thrice by sterile PBS. Following additional 3 min of centrifugation at 1000 r/min, DMEM was added to prepare cells into single cell suspensions (1000 cells/mL), which were later added into the 35-mm low-attachment dish (1 mL each) and cultivated with serum-free condition under 37 °C with 5% CO_2_ for a 21-day period, with medium change at 3-day intervals. At last, the inverted microscope was utilized to count sphere number (diameter, > 50 mm).

### Quantitative real-time polymerase chain reaction (qRT-PCR)

Total RNA was extracted from tissue lysates using TRIzol reagent and reverse transcribed with a commercial kit (Takara, Dalian, China). Quantitative PCR was performed using SYBR Premix EX Taq Master Mix (Takara) on an iCycler Real-Time System (Bio-Rad) with 40 cycles of 95 °C for 15 s, 95 °C for 5 s, and 60 °C for 30 s. All primers were synthesized by Sangon Biotech (Shanghai, China) with the following sequences: miR-641, sense 5’-GGCAGCAAGTCATCATTCCA-3’ and antisense 5’-GGCTGGTGATGCAGGAATTC-3’; U6, sense 5’-CATGTACGTTGCTATCCAGGC-3’ and antisense 5’-CTCCTTAATGTCACGCACGAT-3’. Relative miR-641 expression was normalized to U6 and calculated via the 2^−ΔΔCt^ method.

### Dual luciferase reporter gene assay

The TargetScan database predicted a miR-641 binding site in the TMEFF2 3’-UTR. Both wild-type (WT) and mutant (MUT) versions of this region were synthesized by Genechem (Shanghai) and cloned into the pGL3 vector (Promega, Madison, WI, USA). HEK293T cells were co-transfected with either the WT or MUT reporter vector along with miR-641 mimics or control mimic using Lipofectamine 2000 (Thermo Fisher Scientific, Waltham, MA, USA). After 48-hour incubation, luciferase activity was measured using the Dual-Luciferase Assay System (Promega, Madison, WI, USA).

### RNA immunoprecipitation (RIP) assay

To verify direct miR-641-TMEFF2 mRNA binding, we performed RNA immunoprecipitation (RIP) using ‌5 µg anti-AGO2 antibody (Millipore, 07–1100)‌ with ‌normal mouse IgG (Millipore, 12–371)‌ as control. After overnight incubation and Protein G bead capture, co-precipitated RNAs were analyzed by qRT-PCR. Target enrichment was quantified via 2^−ΔΔCt^ method using TMEFF2/miR-641-specific primers, with three biological replicates.

### Western blotting

Whole tissue and cell lysates were prepared using RIPA buffer, with protein concentration determined by a BCA kit (Beyotime). After separation via 10% SDS-PAGE and transfer to PVDF membranes, blots were blocked with 5% milk and incubated overnight at 4 °C with specific primary antibodies from Abcam: anti-PCNA (ab92552), anti-CD133 (ab222782), anti-CD44 (ab254530), anti-OCT4 (ab181557), anti-TMEFF2 (ab271123), P-MEK (ab307509), MEK (ab33918), P-PI3K (ab278545), PI3K (ab154598), and anti-β-actin (ab8227). Following incubation with HRP-conjugated secondary antibody (Abcam, ab6721), protein bands were visualized using an ECL kit (Beyotime), with band intensities quantified by ImageJ software relative to β-actin.

### In vivo xenograft tumor assay

Eighteen 4-week-old BALB/c nude mice were procured from Yujian Biotechnology Co., Ltd (Hangzhou, China) and housed under controlled conditions (22 °C, 12-h light/dark cycle) with free access to food and water. The study protocol received approval from the Animal Ethics Committee of Affiliated Hospital 6 of Nantong University(YCNA-0134-JK1). Pancreatic cancer cells transfected with NC, miR-641 inhibitor, or miR-641 mimic were resuspended in PBS (1 × 10⁶ cells/100 µL) and subcutaneously injected into the right rear flank. Tumor dimensions were measured weekly using the formula 0.5 × length × width². After 28 days, euthanasia was performed via 5% isoflurane overdose followed by cervical dislocation, in accordance with standard protocols where anesthesia was administered using isoflurane inhalation prior to invasive procedures, and euthanasia could also involve cervical dislocation or CO_2_ asphyxiation to meet AVMA guidelines for humane endpoints. Excised xenograft tumors were weighed, and TMEFF2 expression levels were analyzed by immunohistochemistry (IHC).

### Statistical analysis

All experiments were performed in triplicate. Data are presented as mean ± standard deviation and analyzed using SPSS 20.0. Statistical comparisons were made with paired Student’s t-test (between groups), one-way ANOVA with Tukey’s post hoc test (among multiple groups). Survival analysis was performed using Kaplan-Meier curves with log-rank test. Statistical significance was set at *P* < 0.05.

## Results

### High miR-641 expression is observed within pancreatic cancer tissues and cell lines

**Fig. 1:**
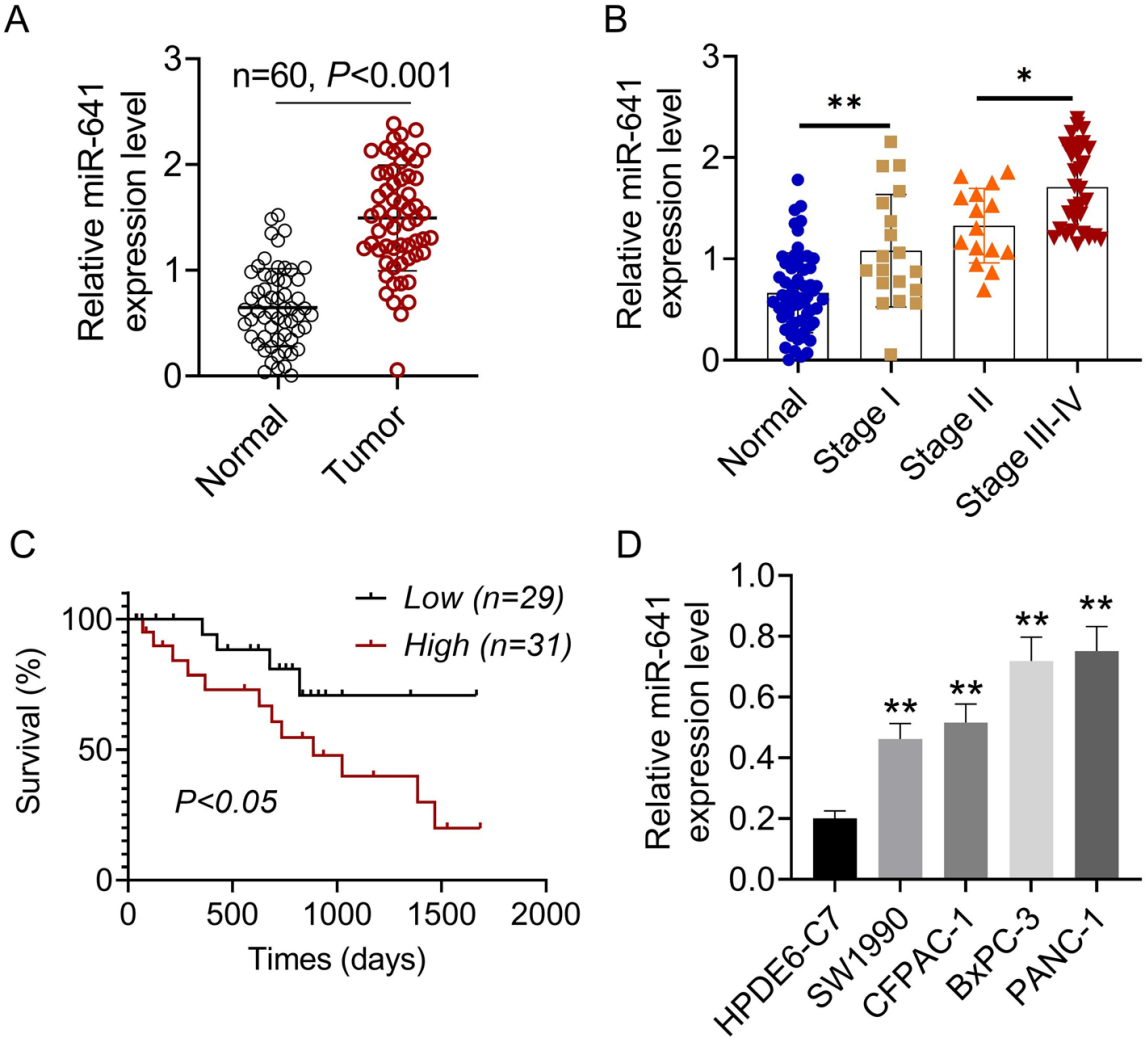
The expression of miR-641 in pancreatic cancer. **A** The expression of miR-641 in 60 paired pancreatic cancer tumor tissue and non-tumor tissue was explored by qPCR. **B** The expression of miR-641 in different stages of pancreatic carcinoma patients. *P < 0.05, **P < 0.01. **C** Kaplan–Meier analysis showed that higher expression of miR-641 predicted poor survival rate of pancreatic cancer patients. **D** The expression of miR-641 in pancreatic cancer cells (SW1990, CFPAC-1, BxPC-3 and PANC-1) and normal human pancreatic ductal epithelial cells (HPDE6-C7) was examined by qRT-PCR. **P < 0.01 vs. HPDE6-C7. Data was shown as mean ± SD

MiR-641 expression in clinical samples was also determined by qPCR. As discovered, miR-641 expression markedly increased within tumor tissue relative to normal tissue (*P* < 0.001, Fig. 1). Table 1 revealed the relationship between miR-641 expression and clinical features of pancreatic cancer patients. We can see that miR-641 expression was correlated with the pancreatic cancer distant metastasis and TNM stage.

**Table 1:** Relationship between miR-641 expression and clinical features of PC patients

Characteristics	Number of patients	MiR-641 Low expression (< median)	MiR-641 High expression (≥median)	*P* value
Number	60	28	32	
Ages(years)				0.507
< 60	29	14	15	
≥ 60	31	14	17	
Gender				0.480
Female	27	12	15	
Male	33	16	17	
Lymphatic metastasis				0.219
T1 + T2	30	12	18	
T3 + T4	30	16	14	
N classification				0.103
N0	28	16	12	
N1-N2	32	12	20	
Distant metastasis				0.037
M1	32	11	21	
M0	28	17	11	
TNM stage				0.020
I-II	29	18	11	
III-IV	31	10	21	

With the development of pancreatic carcinoma, the relative miR-641 expression was increased, which was higher expressed in stage III-IV than that in stage I and II (Fig. 1B). Moreover, Kaplan-Meier analysis showed that higher expression of miR-641 predicted poor survival rate of pancreatic cancer patients (*P* < 0.05, Fig. 1C). The expression of miR-641 in pancreatic cancer cells (SW1990, CFPAC-1, BxPC-3 and PANC-1) and normal human pancreatic ductal epithelial cells (HPDE6-C7) was examined by qRT-PCR. The results showed that miR-641 was overexpressed in SW1990, CFPAC-1, BxPC-3 and PANC-1 cell lines compared with that in HPDE6-C7 cells (*P* < 0.01, Fig. 1D).

### miR-641 down-regulation inhibits pancreatic cancer cell growth and stem cell characteristics

**Fig. 2:**
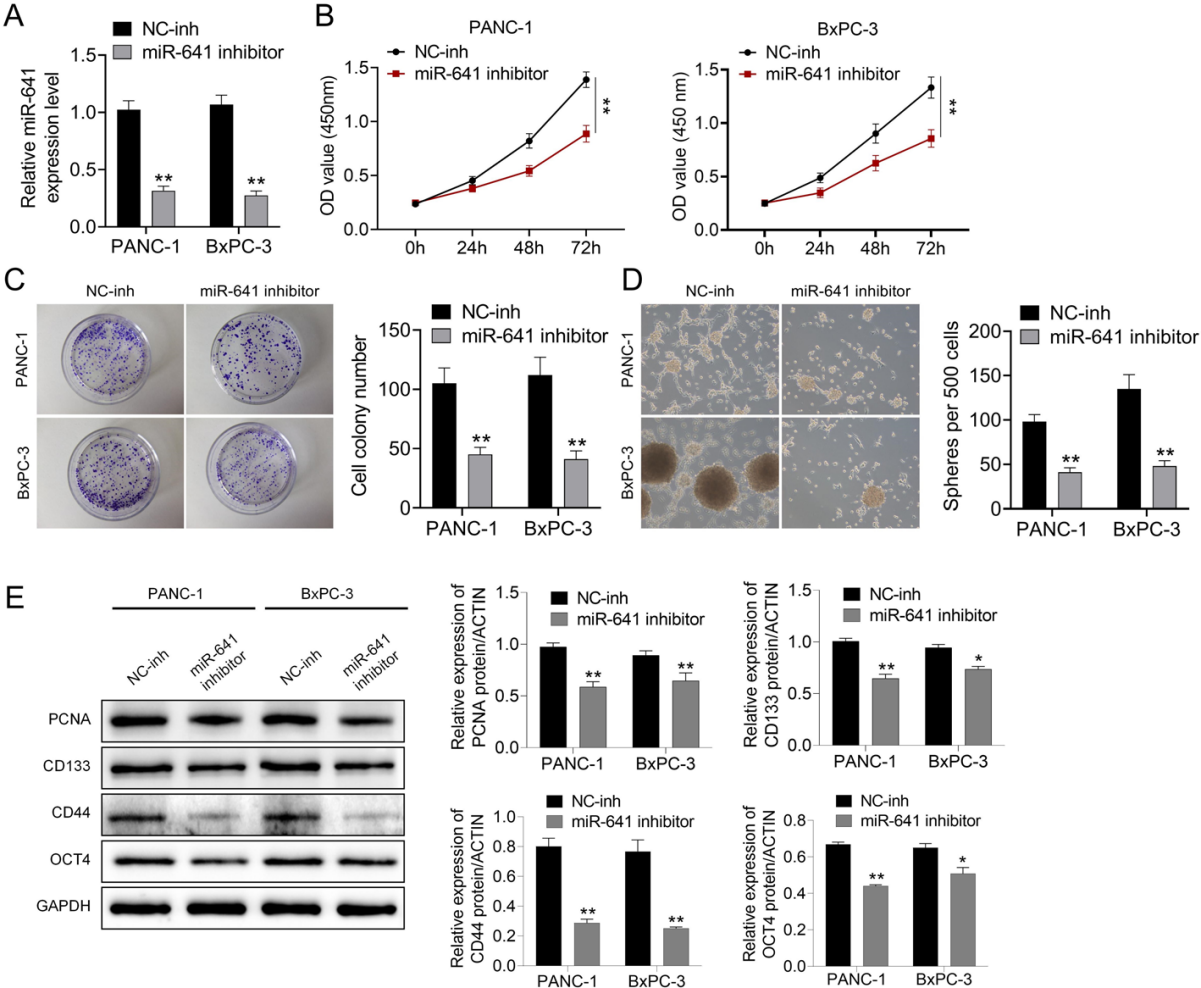
Down-regulation of miR-641 inhibited cell proliferation and stem cell characteristics of pancreatic cancer cells.**A**.Pancreatic cancer cells (PANC-1 and BxPC-3) were transfected with miR-641 inhibitor, and miR-641 expression was examined by RT-PCR. **B**. Cell proliferation was examined by CCK8. **C–D** Cell cloning and spheroidization ability of PANC-1 and BxPC-3 cells was tested. **E**. The expression of PCNA, CD133, CD44, and OCT4 in PANC-1 and BxPC-3 cells was examined by western blot. Data was shown as mean ± SD. **P < 0.01 vs. NC-inh

For exploring the function of miR-641 in pancreatic cancer cell proliferation and stem cell characteristics, PANC-1 and BxPC-3 cells underwent transfection using NC-inh and miR-641 inhibitor. From Fig. 2A, miR-641 expression decreased within PANC-1 and BxPC-3 cells. Cell proliferation was examined through CCK8 assay. It was shown that down-regulation of miR-641 remarkably suppressed the growth of these two cell lines (Fig. 2B, **P < 0.01). The miR-641 inhibitor suppressed cell cloning and spheroidization capacities in these cells (Fig. 2C**–**D, **P < 0.01). Meanwhile, expression of PCNA, CD133, CD44, and OCT4 in these cells was examined through Western blotting. As shown in Fig. 2E, the miR-641 inhibitor inhibited PCNA, CD133, CD44, and OCT4 levels within PANC-1 and BxPC-3 cells (Fig. 2E, **P < 0.01).

### Prediction of miR-641 targeting TMEFF2

**Fig. 3:**
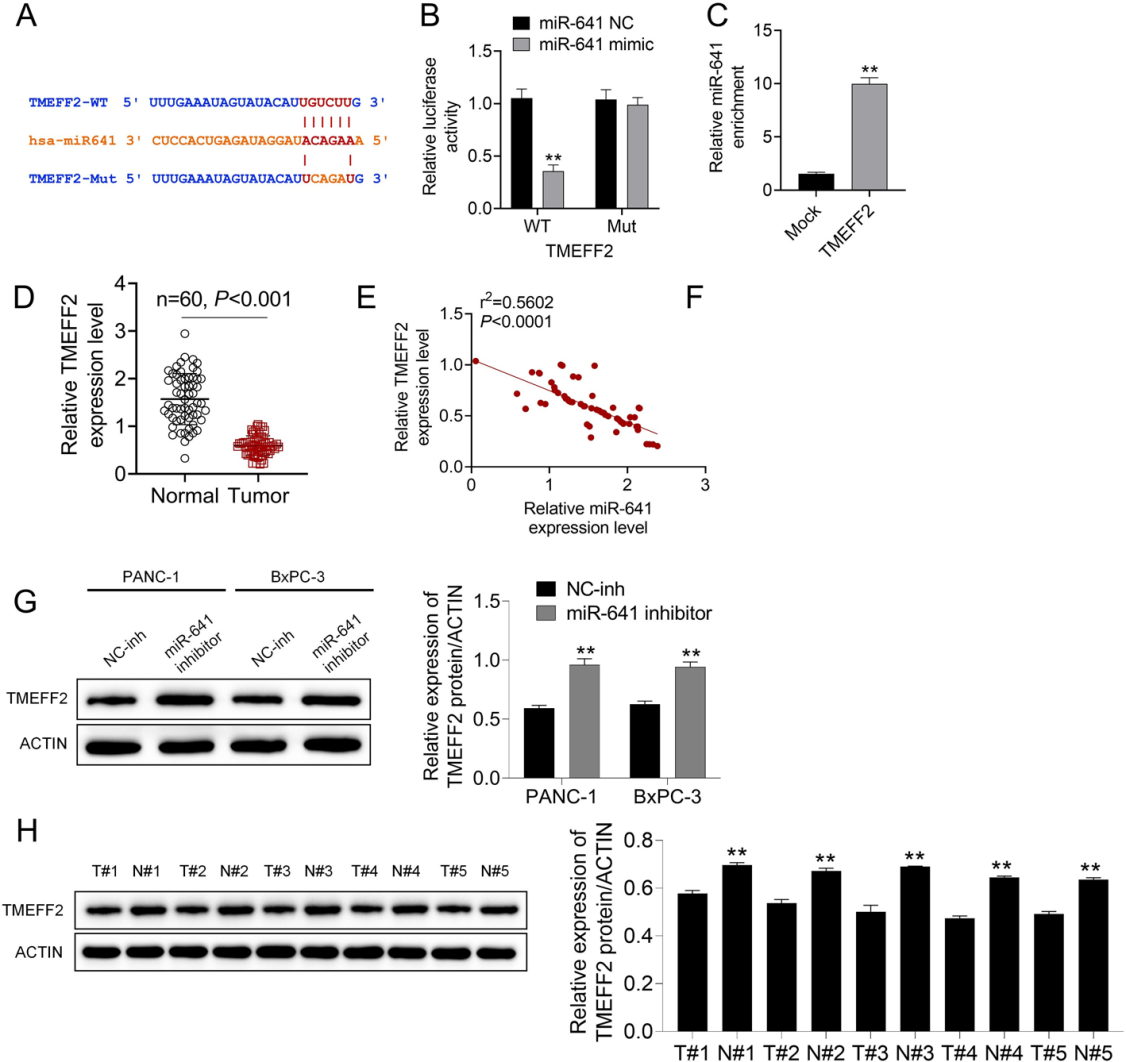
Prediction of miR-641 targeting TMEFF2. **A** Bioinformatics analysis of TMEFF2 targeting miR-641. **B–C**. The luciferase reporter gene experiment and RIP experiment were performed. **D**. The mRNA expression of TMEFF2 in pancreatic cancer tissue and non-tumor tissue was detected by qPCR. **E**. The expression of TMEFF2 mRNA displayed a negative correlation with miR-641 expression in 60 pancreatic cancer samples. **F**. The protein expression of TMEFF2 in PANC-1 and BxPC-3 cells transfected with miR-641 inhibitor was examined by western blot. **G**. The protein expression of TMEFF2 in paired pancreatic cancer tissue and non-tumor tissue was tested by western blot. Data was shown as mean ± SD. **P < 0.01 vs. NC-inh

We further explored the targets of miR-641. By bioinformatics analysis, TMEFF2 was possibly the targeted mRNA of miR-641 (Fig. 3A). As confirmed by luciferase reporter gene experiment and RIP experiment, miR-641 showed the direct targeting relation with TMEFF2 (Fig. 3B–C). The TMEFF2 mRNA expression within pancreatic cancer and non-tumor tissues was then studied. Based on the results, TMEFF2 showed down-regulation within pancreatic cancer samples (***P < 0.001, Fig. 3D). Moreover, TMEFF2 mRNA expression displayed a negative correlation with miR-641 expression in 60 pancreatic cancer samples (r^2^ = 0.5602, *P* < 0.0001, Fig. 3E). The TMEFF2 protein expression levels within PANC-1 and BxPC-3 cells after miR-641 inhibitor transfection were then examined through Western blotting, as a result, miR-641 inhibitor enhanced TMEFF2 protein level (**P < 0.01, Fig. 3F). TMEFF2 protein levels within paired pancreatic cancer and non-tumor tissues were tested through Western blotting. The results exhibited that TMEFF2 was down-regulated in pancreatic cancer tissue (**P < 0.01, Fig. 3G).

### MiR-641 targeting TMEFF2 regulated pancreatic cancer cell growth and stem cell characteristics

**Fig. 4:**
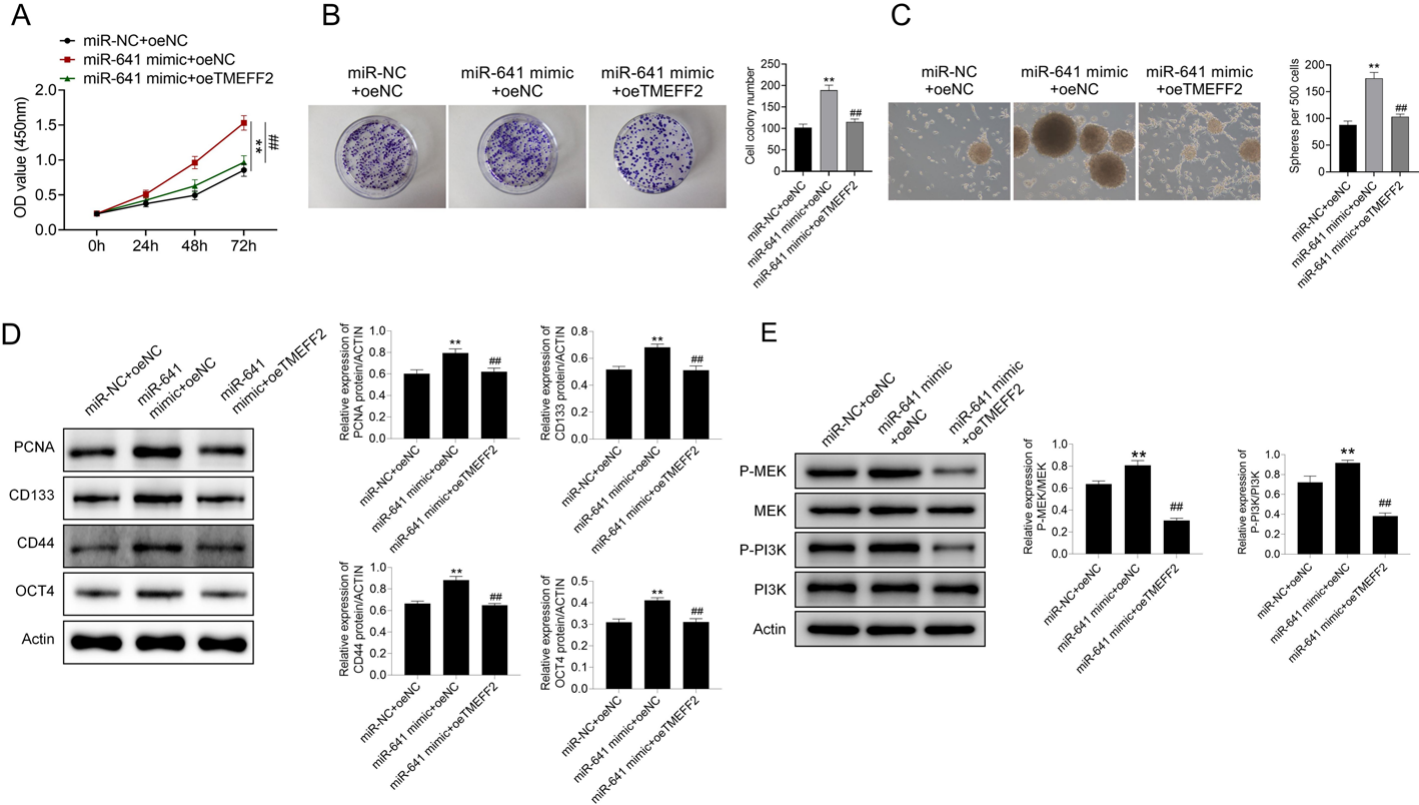
MiR-641 targeting TMEFF2 regulated cell proliferation and stem cell characteristics of pancreatic cancer cells PANC-1 cells were transfected with miR-NC+oeNC, miR-641 mimic+oeNC, or miR-641 mimic+oeTMEFF2. **A–C**. Cell proliferation, cell cloning and cell spheroid formation. **D**. The expressions of PCNA, CD133, CD44, and OCT4 in PANC-1 cells with miR-NC+oeNC, miR-641 mimic+oeNC, or miR-641 mimic+oeTMEFF2 transfections were detected by western blot. Data was shown as mean ± SD. **P < 0.01 vs. miR-NC+oeNC. ^##^*P* < 0.01 vs. miR-641 mimic+oeNC

PANC-1 cells after miR-NC+oeNC, miR-641 mimic+oeNC, or miR-641 mimic+oeTMEFF2 transfection were analyzed for their growth and stem cell characteristics. Clearly, miR-641 mimic enhanced PANC-1 cell growth (*P* < 0.01), while TMEFF2 rescued the role of miR-641 mimic in cell growth (Fig. 4A). MiR-641 mimic also promoted cell cloning and spheroidization ability of PANC-1 cells, which was offset by oeTMEFF2 in PANC-1 cells (Fig. 4B**–**C). The expression of PCNA, CD133, CD44, and OCT4 in PANC-1 cell with miR-NC+oeNC, miR-641 mimic+oeNC, or miR-641 mimic+oeTMEFF2 transfections was detected by western blot. As exhibited in Fig. 4D, miR-641 mimic promoted the expression of PCNA, CD133, CD44, and OCT4, while TMEFF2 showed the opposite effect (Fig. 4D). Moreover, we found that miR-641 mimic promoted the phosphorylation of MEK and PI3K, while TMEFF2 reversed the effect of miR-641 mimic on p-MEK and p-PI3K (Fig. 4E).

### TMEFF2 inhibited pancreatic cancer cell growth and stem cell characteristics via MEK/PI3K signaling

**Fig. 5:**
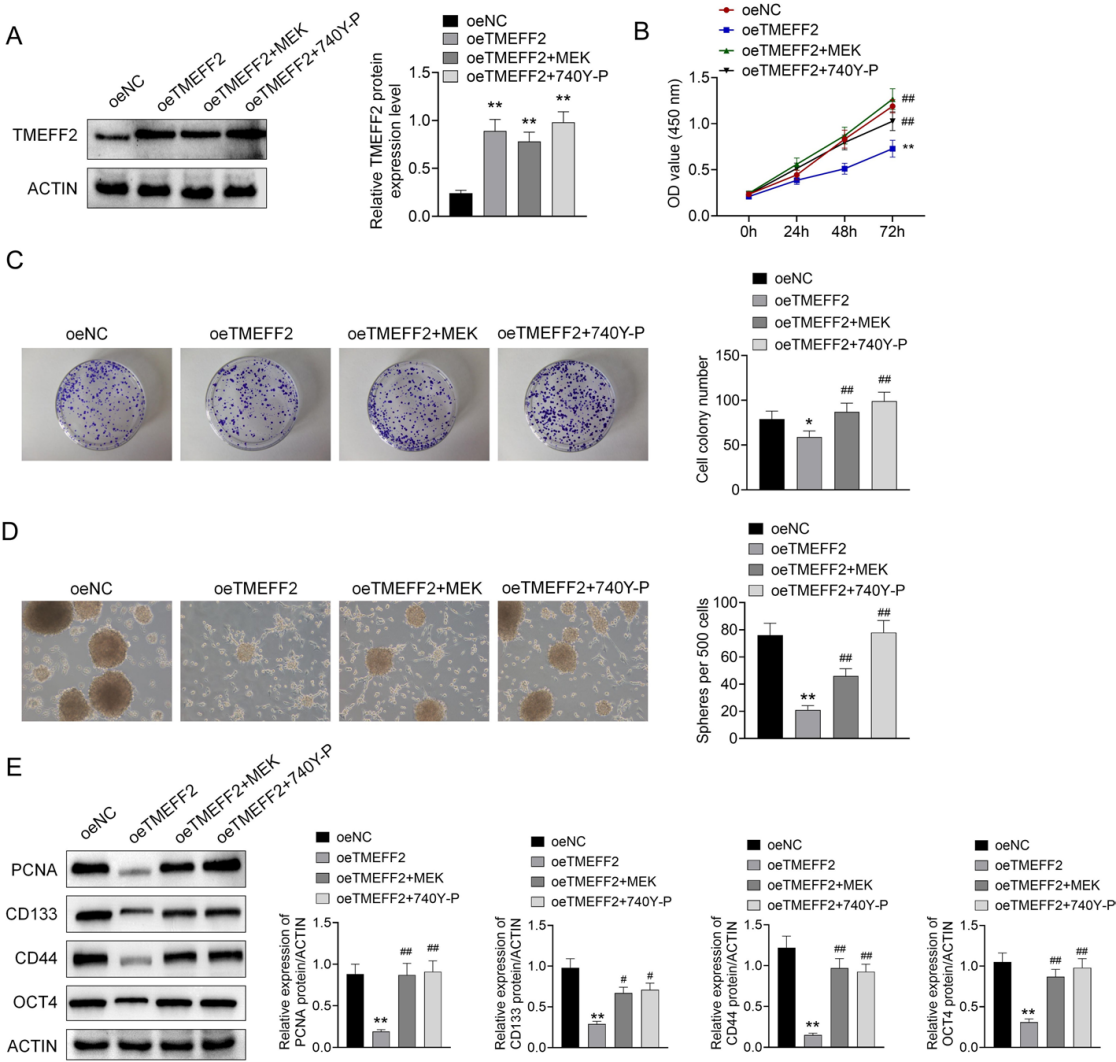
TMEFF2 inhibited pancreatic cancer cell growth and stem cell characteristics via MEK/PI3K signaling PANC-1 cells were treated with TMEFF2-overexpression transfection, and then with MEK-overexpression co-transfection or PI3K agonist (740Y-P) treatment. **A**. The protein expressions of TMEFF2 in oeTMEFF2, oeTMEFF2 + MEK, and oeTMEFF2 + 740Y-P groups were examined by western blot. **B–D**. Cell proliferation, cell cloning and spheroidization ability were detected. **E**. The expressions of PCNA, CD133, CD44, and OCT4 in PANC-1 cells was detected by western blot. Data was shown as mean ± SD.*P < 0.05, **P < 0.01 vs. oeNC. ^##^*P* < 0.01 vs. oeTMEFF2

PANC-1 cells were treated with TMEFF2-overexpression transfection, and then with MEK-overexpression co-transfection or PI3K agonist (740Y-P) treatment. As shown in Fig. 5A, the expressions of TMEFF2 in oeTMEFF2, oeTMEFF2 + MEK, and oeTMEFF2 + 740Y-P groups were significantly increased compared with that in oeNC group. It was observed that TMEFF2 overexpression suppressed cell proliferation, cell cloning and spheroidization ability of PANC-1 cells (Fig. 5B, C and D), while MEK overexpression or 740Y-P could reversed the effect of TMEFF2 overexpression. Moreover, TMEFF2 overexpression decreased the expression of PCNA, CD133, CD44, and OCT4, while MEK overexpression or 740Y-P showed the opposite effect (Fig. 5E).

### MiR-641 targeting TMEFF2 promoted tumor growth in vivo

**Fig. 6:**
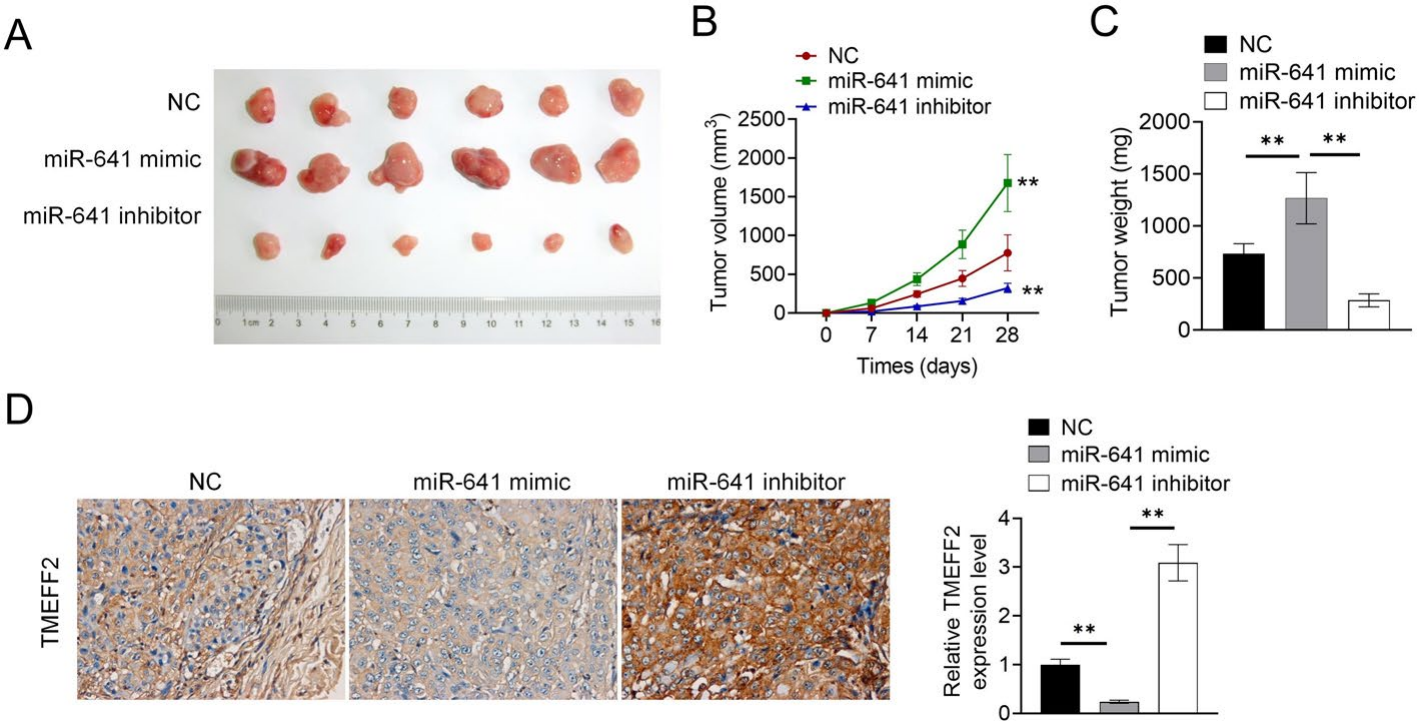
MiR-641 targeting TMEFF2 promoted tumor growth in vivo **A**. The PANC-1 cells of NC, miR-641 mimic, and miR-641 inhibitor were constructed respectively, and then the transplant tumor mice were established to observe tumor morphology. **B**.Tumor growth volume curve was recorded. **C**. Tumor weight was calculated. **D**. IHC was used to detect the expression of TMEFF2. Data was shown as mean ± SD. **P < 0.01

The PANC-1 cells of NC, miR-641 mimic, and miR-641 inhibitor were constructed respectively, and then the transplant tumor mice were established to observe tumor volume and size. We performed IHC for detecting TMEFF2 expression. From Fig. 6A, B and C, miR-641 mimic promoted tumor development and increased tumor weight (*P* < 0.01), miR-641 inhibitor had opposite effects (*P* < 0.01), as compared with the NC treatment. Moreover, the miR-641 mimic inhibited the expression of TMEFF2 (Fig. 6D). The miR-641 inhibitor promoted the expressions of TMEFF2 (Fig. 6D).

## Discussion

The impact of miRNA on pancreatic cancer occurrence and development involves multiple aspects. Firstly, some certain miRNAs, like miR-145 and miR-103, may influence various processes like cancer occurrence, growth and differentiation, infiltration and metastasis by regulating downstream target genes [18]. These miRNAs may play the role as oncogenes and tumor suppressors, depending on their regulation of target genes. Secondly, miRNA can enhance cancer cell proliferation and metastasis through influencing glycolysis. The glycolysis process in pancreatic cancer cells produces excessive substrates and accelerates tumor cell proliferation and migration via the interaction between glycolytic core enzymes and actin [19]. The core enzymes and intermediate products of aerobic glycolysis can participate in epithelial mesenchymal transition, angiogenesis, and proliferation signal transduction or epigenetic regulation, thereby influencing pancreatic cancer growth and metastasis [20]. Additionally, aberrant miRNA expression is probably associated with pancreatic cancer invasion and metastasis. To take an example, miR-145 can target and inhibit the translation of Ang-2 by acting on siRNA of Ang-2 in pancreatic cancer cells, and reduced Ang-2 expression can slow down the progression of the neovascular system [21, 22]. Overall, although the specific mechanism of action of miRNAs in pancreatic cancer is still not fully understood, they provide new possible avenues for diagnosing and treating pancreatic cancer. Through further research, we can better understand the impact of miRNA on pancreatic cancer, providing more clues and ideas for future diagnosis and treatment.

MiR-641 has a complex effect on tumors, which involves multiple aspects [15, 23]. For one thing, miR-641 expression can increase or decrease in different cancer cells, participating in different biological events like cell growth, apoptosis, migration and invasion, while playing a regulatory function similar to that of oncogenes or tumor suppressors [24, 25]. This effect may be related to the mutual influence of multiple target genes. For the other thing, some studies have shown that miR-641 may serve as a new target with potential value in cancer diagnosis, drug resistance, treatment, and other aspects. For instance, miR-641 may accelerate tumor cell growth and migration through affecting glycolysis. Additionally, abnormal miR-641 expression is probably associated with pancreatic cancer migration and invasion [26]. However, the mechanism underlying miR-641 in tumors remains largely unclear, which deserves more investigations, so as to offer more evidence and ideas for future diagnosis and treatment.

The TMEFF2 gene, also known as TPEF, Tomoregulin, and HPP1, is a protein-coding gene with FS-like and epidermal growth factor (EGF)-like domains [27]. The TMEFF2 gene has a key effect on different physiopathological processes, like embryogenesis, neural development, neuroprotection, and tumorigenesis. First, the TMEFF2 gene-encoded transmembrane protein contains the EGF-like functional domain, two follistatin-like functional domains, and a transmembrane region. These domains can bind to various molecules, such as transforming growth factor-β family (TGF-β superfamily), platelet-derived growth factor (PDGF), as well as vascular endothelial growth factor (VEGF), and inhibit their receptor activation [28, 29]. Secondly, the TMEFF2 gene has different roles in various cancers. For example, for endometrial and prostate cancers, TMEFF2 is upregulated, while within cancers like colorectal, pancreatic and gastric cancers, high methylation of CpG island in TMEFF2 gene promoter often occurs, leading to downregulation of TMEFF2 expression [30]. This indicates that TMEFF2 may become a tumor specific or methylated biomarker with broad application prospects. However, the exact physiological function of TMEFF2 and its mechanism of action in tumors are still not fully understood. Studies have shown that TMEFF2 may have anti-tumor effects, such as longer survival in patients expressing TMEFF2 in gastric cancer [31]. This suggests that TMEFF2 may have the ability to inhibit cancer. In addition, experiments have shown that the extracellular region of TMEFF2 can enhance dopaminergic neuron survival in soluble form. The TMEFF2 gene exerts a key effect on different physiopathological processes, particularly during tumor occurrence and progression. In the present study, we proved that TMEFF2 inhibited pancreatic cancer cell growth and stem cell characteristics via MEK/PI3K signaling. However, more investigations are warranted for clarifying the exact physiological function of TMEFF2 as well as the underlying mechanism in tumors. The present work reported miR-641 up-regulation within pancreatic cancer samples. The cohort size (*n* = 60) may limit statistical power, and future studies with larger samples are warranted to confirm these findings. Pancreatic cancer cells showed higher expression of miR-641 compared with normal cells. MiR-641 down-regulation inhibited cell growth, clone forming ability, invasion and migration in pancreatic cancer cells. Down-regulation of miR-641 inhibited the capability of sphere-forming, reduced CD133 + and CD44 + cell quantities, and suppressed CD133, CD44, and Oct4 expression in pancreatic cancer cells. MiR-641 targeted TMEFF2 for promoting pancreatic cancer cells’ stem cell characteristics. Moreover, miR-641 targeting TMEFF2 promoted tumor growth in vivo, but subcutaneous xenografts have limitations in mimicking the complex pancreatic tumor microenvironment.

This study has the following limitations: (1) TCGA database and limited clinical samples may not fully represent pancreatic cancer subtype heterogeneity, and lack long-term follow-up data to validate the prognostic predictive value of miR-641; (2) Although the involvement of the TMEFF2/MEK/PI3K pathway was confirmed, it remains unclear whether miR-641 coordinately regulates stemness characteristics through epigenetic modifications or alternative targets; (3) Only the subcutaneous xenograft model was employed, failing to simulate the pancreatic cancer microenvironment (e.g., orthotopic or humanized mouse models), and metastatic potential was not evaluated. The priorities for future research are: (1) Integrating multi-center cohorts with single-cell sequencing to analyze the association between miR-641 expression profiles and clinical stages across pancreatic cancer subtypes; (2) Identifying secondary targets of miR-641 via CRISPR screening or proteomics, and exploring their interaction networks with TMEFF2; (3) Developing a miR-641-targeting nanodelivery system to evaluate its synergistic therapeutic effects and resistance mechanisms combined with MEK/PI3K inhibitors in orthotopic models.

In conclusion, miR-641 acts as an oncogene that promotes pancreatic cancer cell growth, invasion as well as stem-cell-like features, which is realized by regulating the expression of TMEFF2. Our findings uncover the interaction mechanism between miR-641 and TMEFF2, providing the new perspective for understanding the pancreatic cancer pathogenesis. The expression levels of miR-641 and TMEFF2 are the candidate biomarkers for diagnosing and assessing the prognosis of pancreatic cancer.

## Supplementary Information

Supplementary Material 1.

## Data Availability

No datasets were generated or analysed during the current study. Additional article data may be obtained by contacting the corresponding author, provided a valid justification is presented.

## Declarations

### Clinical trial statement

The protocol was approved by ethics committee of Affiliated Hospital 6 of Nantong University in accordance with the Declaration of Helsinki (No. YCN-0020–14 J).
