# Supplementary material for: MiR-641 targets TMEFF2/MEK/PI3K to promote stem cell characteristics of pancreatic cancer cells
Source: Discov Oncol. 2026 Feb 7;17:408. doi: 10.1007/s12672-026-04584-2 (PMC12976288; doi:10.1007/s12672-026-04584-2)
Supplement: Supplementary file 1 — Supplementary Material 1. [file 12672_2026_4584_MOESM1_ESM.pdf]

Figure 2

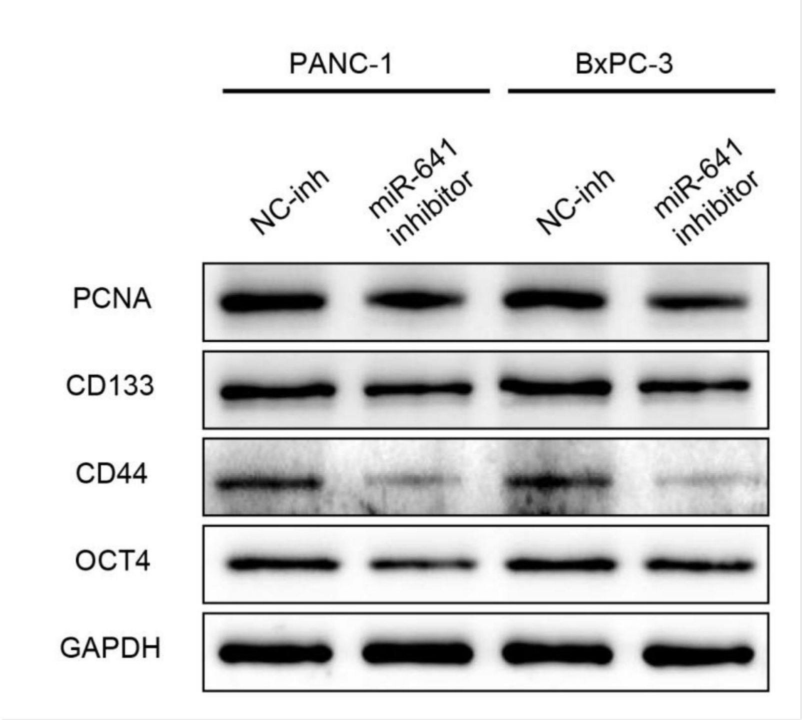

PCNA
29kDa

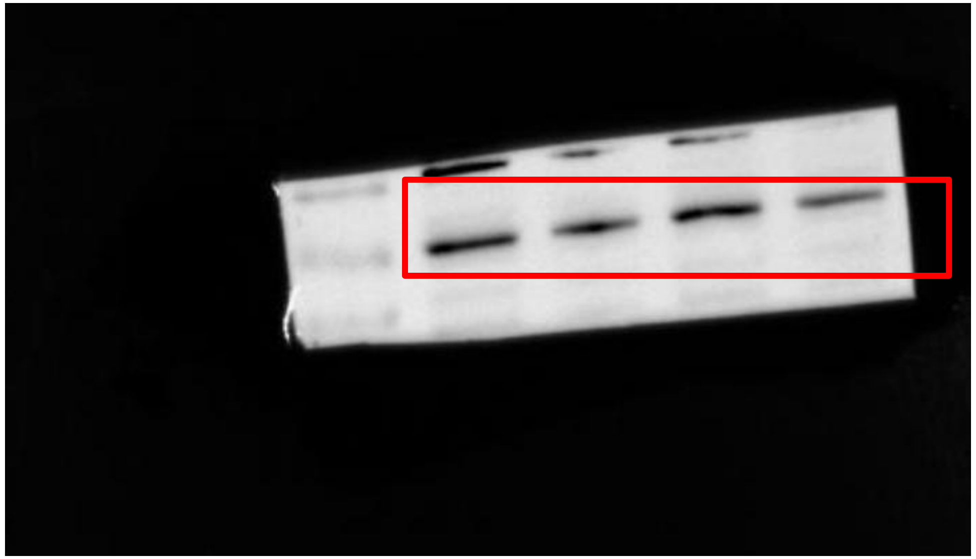

Figure 2

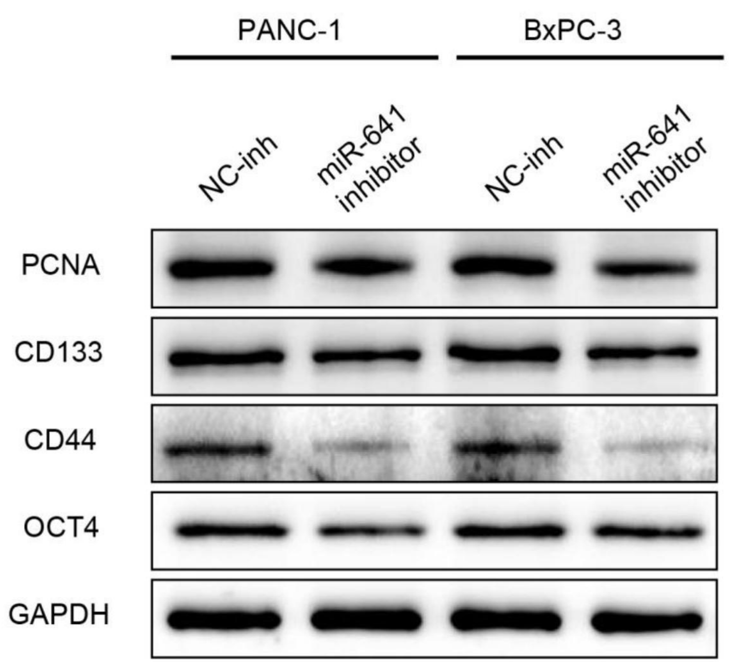

CD133
97kDa

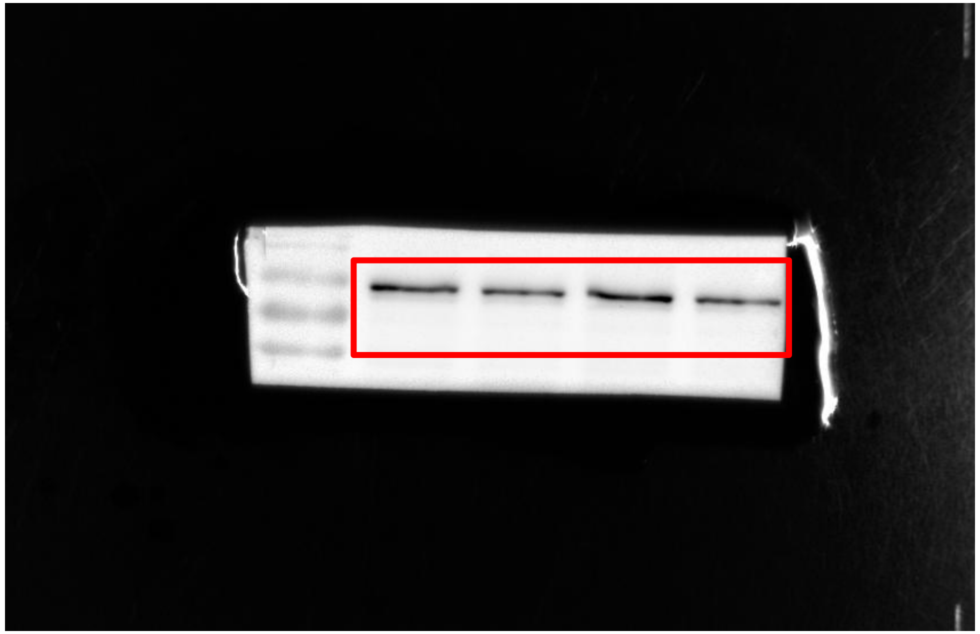

Figure 2

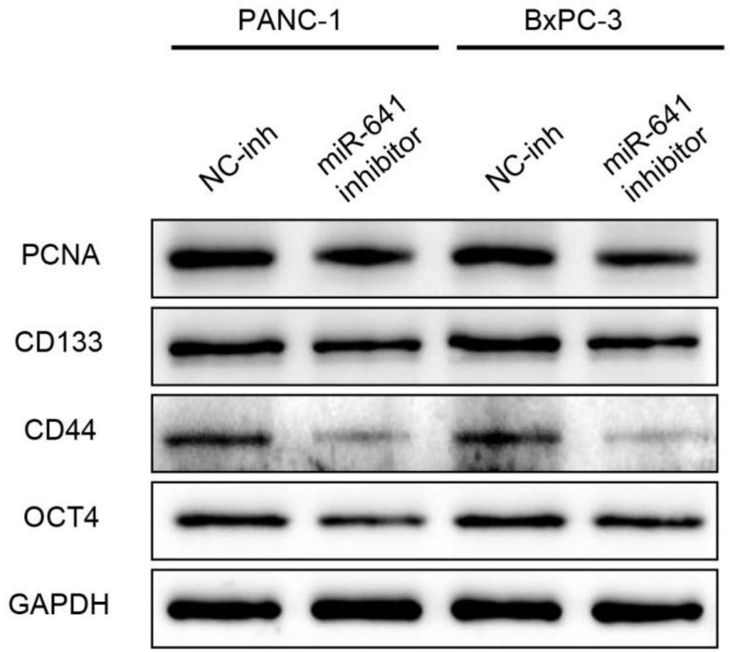

CD44
81kDa

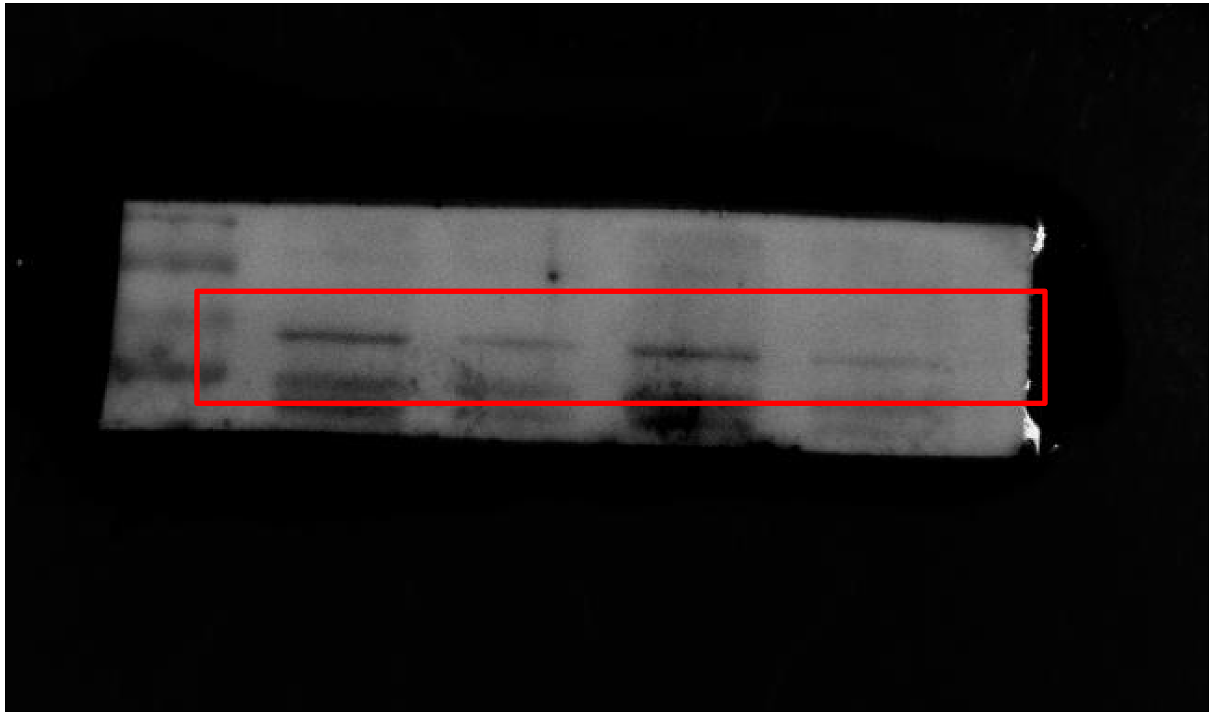

Figure 2

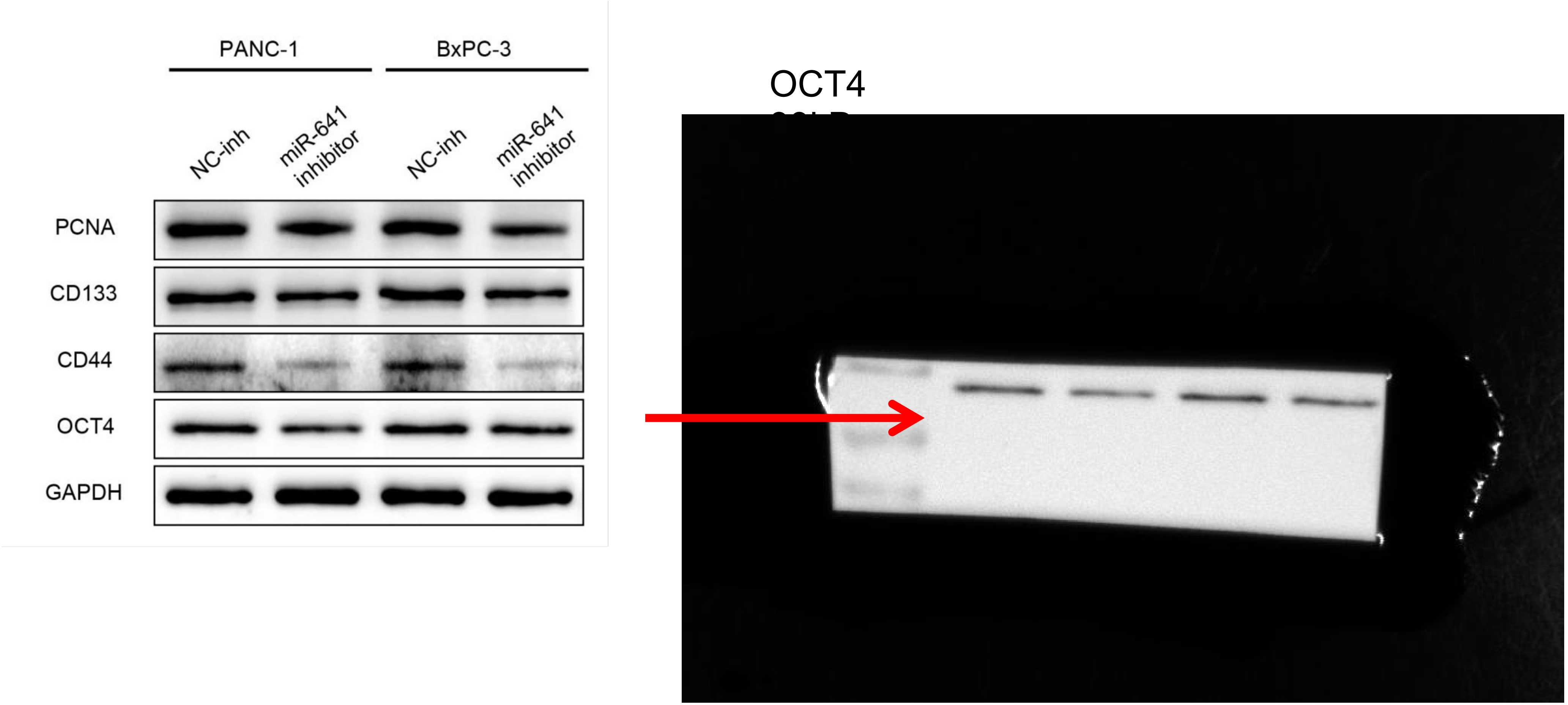

PANC-1
BxPC-3
NC-inh
miR-641 inhibitor
NC-inh
miR-641 inhibitor
PCNA
CD133
CD44
OCT4
GAPDH
OCT4

Figure 2

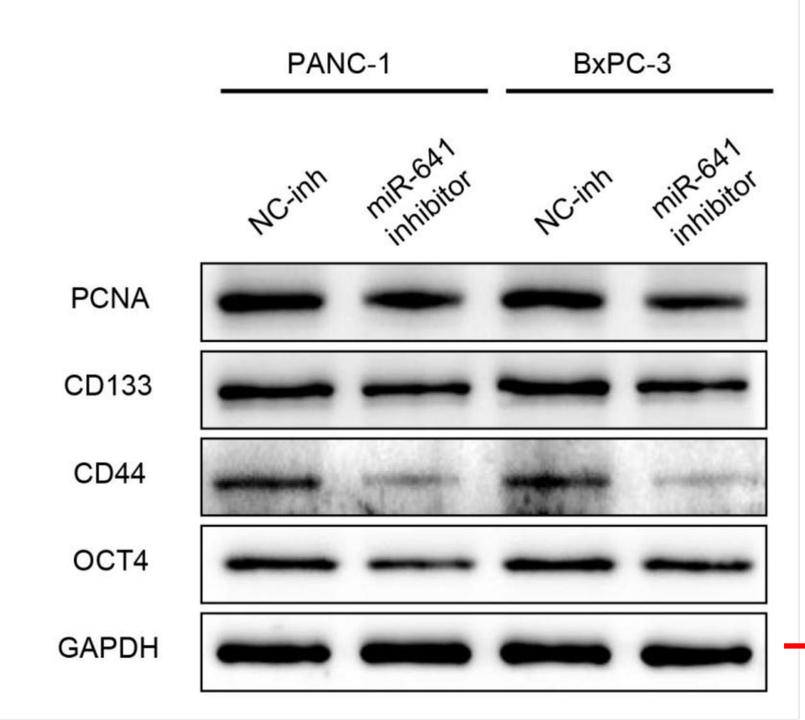

GAPDH
42kDa

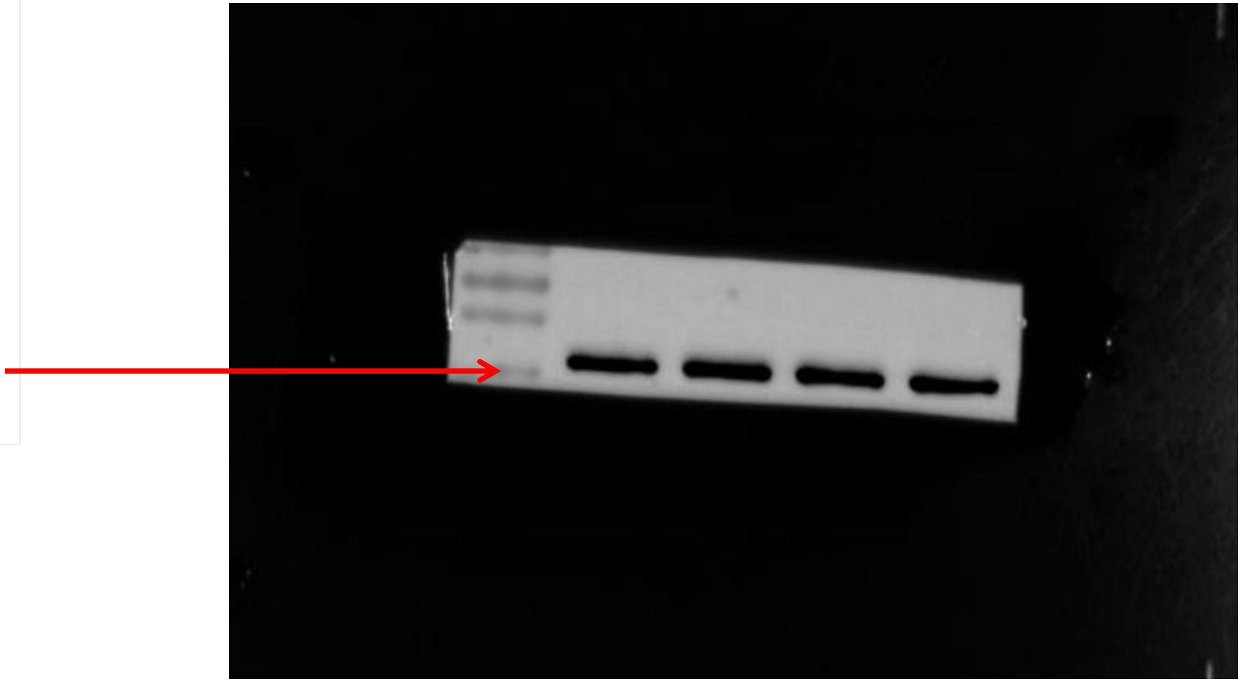

Figure 3F

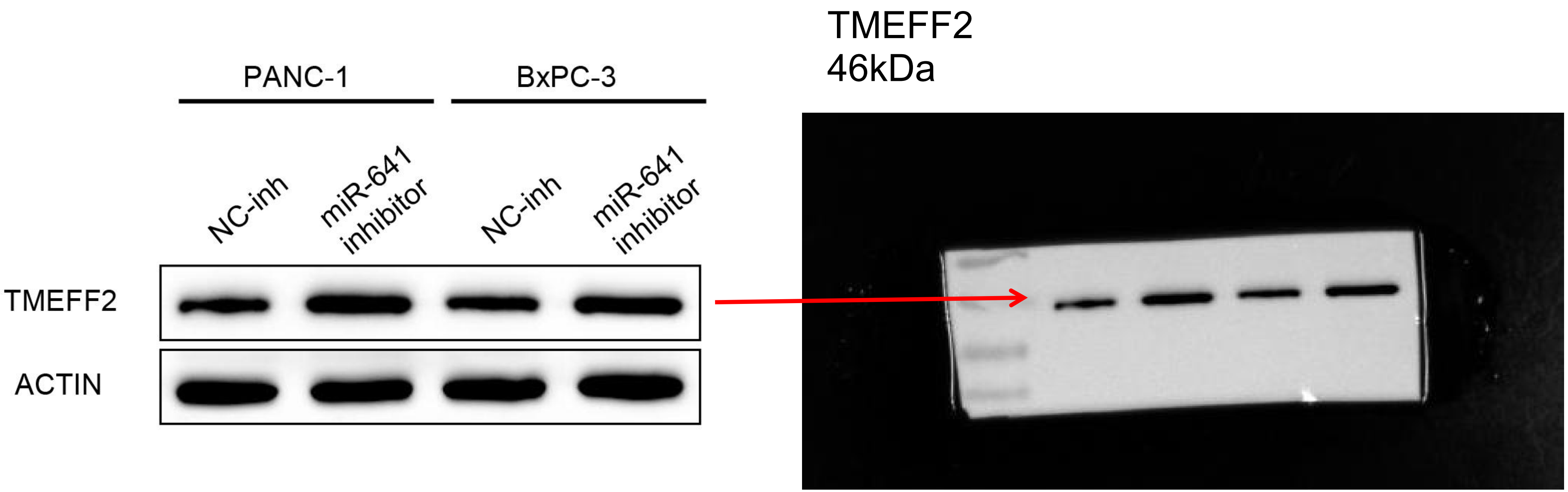
PANC-1
BxPC-3
NC-inh
miR-641 inhibitor
NC-inh
miR-641 inhibitor
TMEFF2
ACTIN
TMEFF2
46kDa

Figure 3F

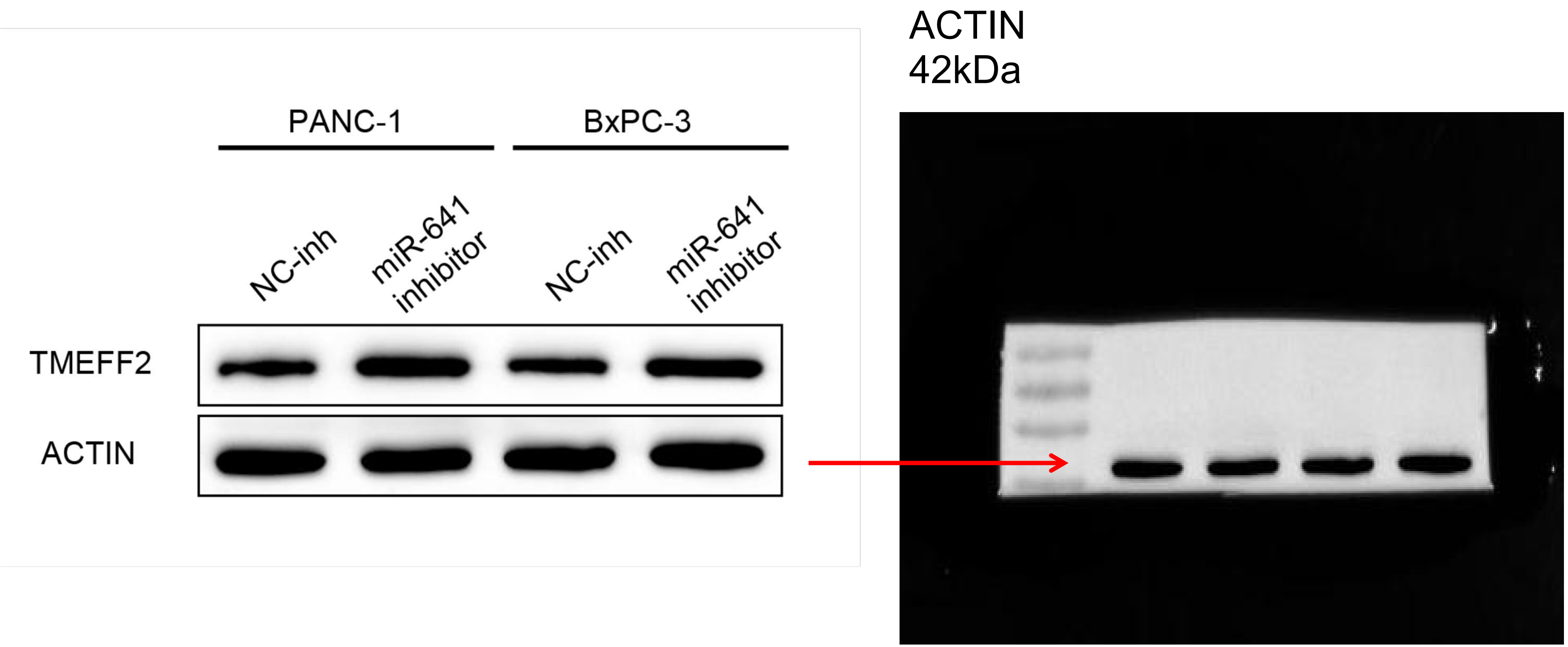

ACTIN
42kDa
PANC-1
BxPC-3
NC-inh
miR-641 inhibitor
NC-inh
miR-641 inhibitor
TMEFF2
ACTIN

Figure 3G

TMEFF2
46kDa

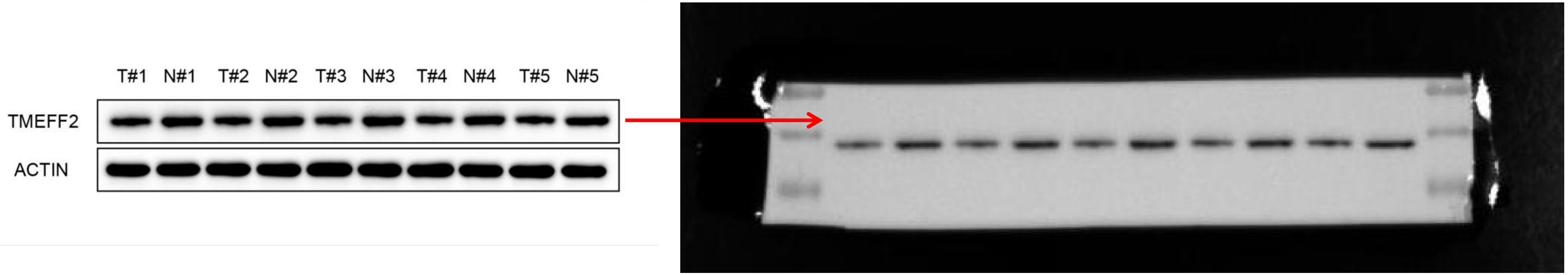

Figure 3G

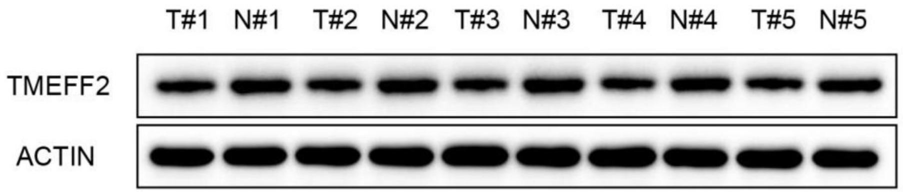

ACTIN
42kDa

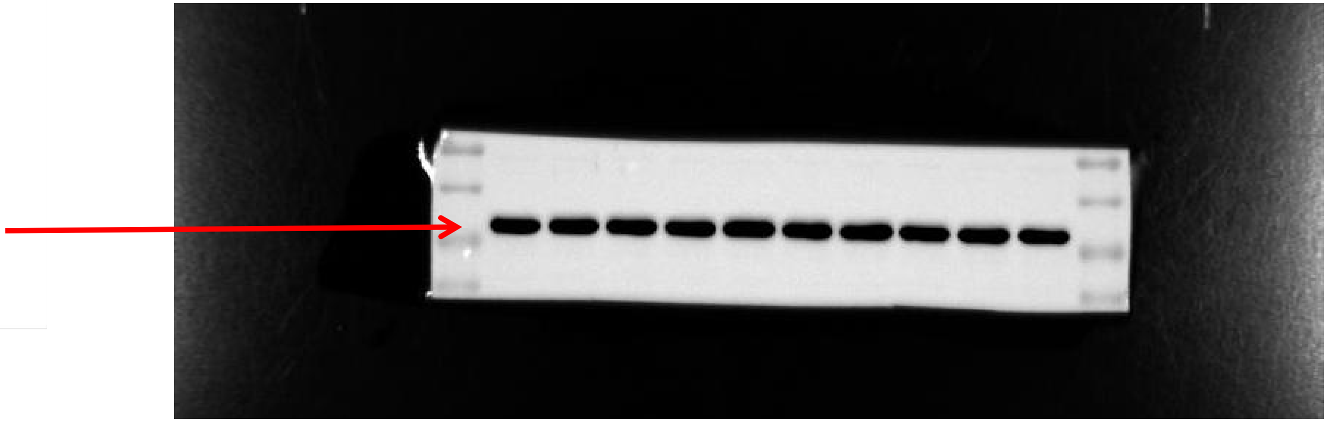

Figure 4E

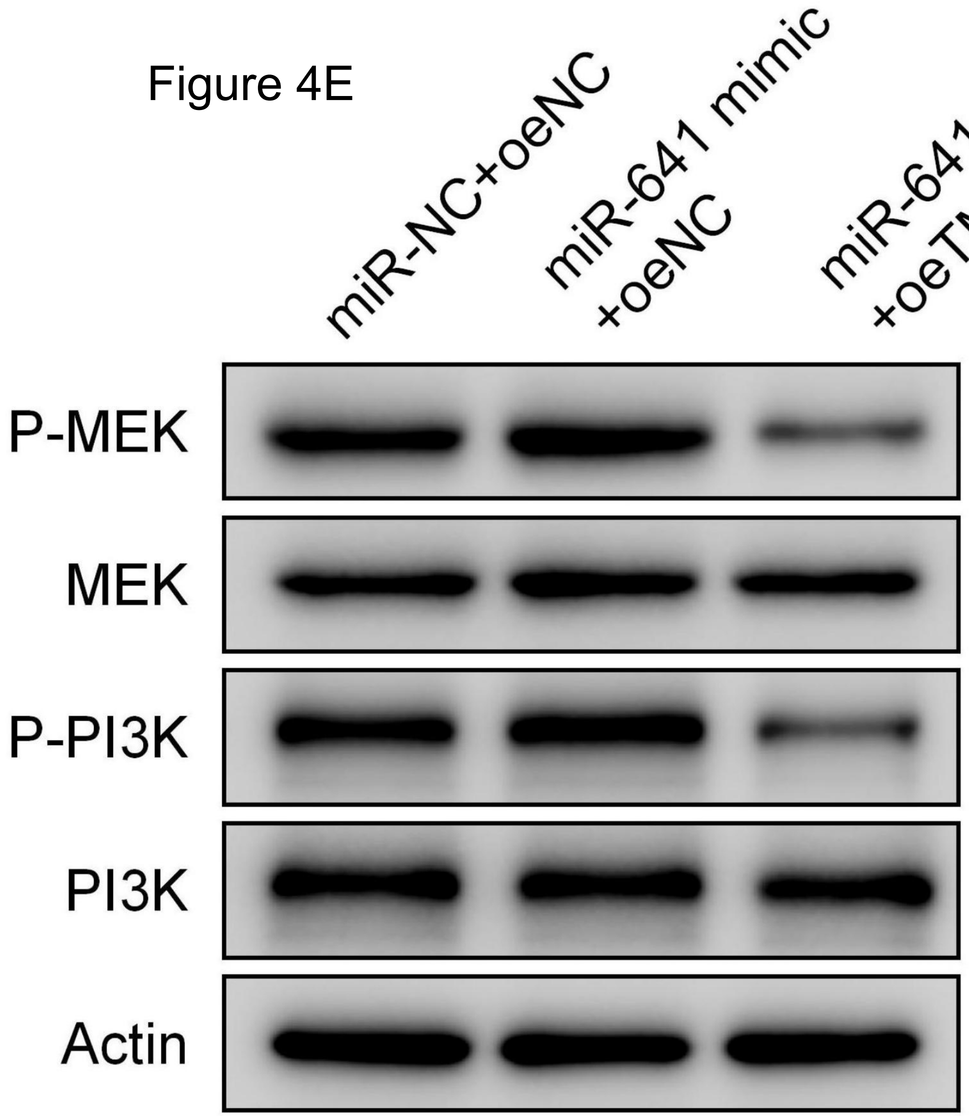

P-MEK
43kDa

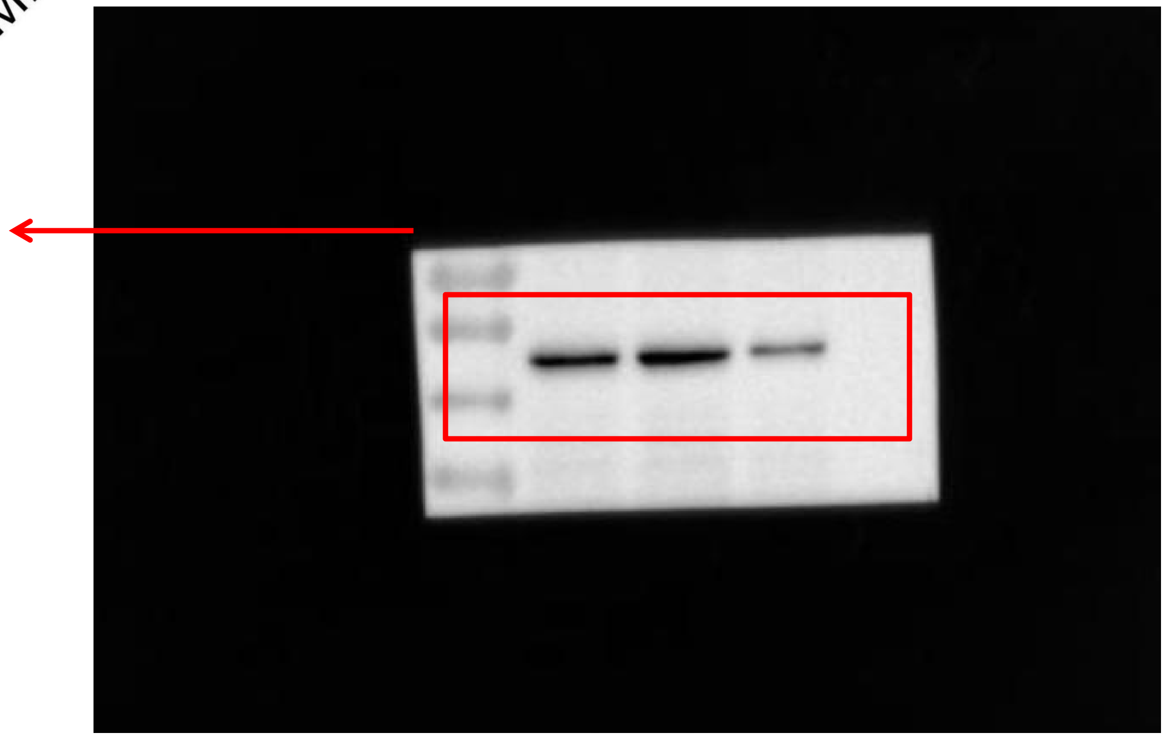

Figure 4E

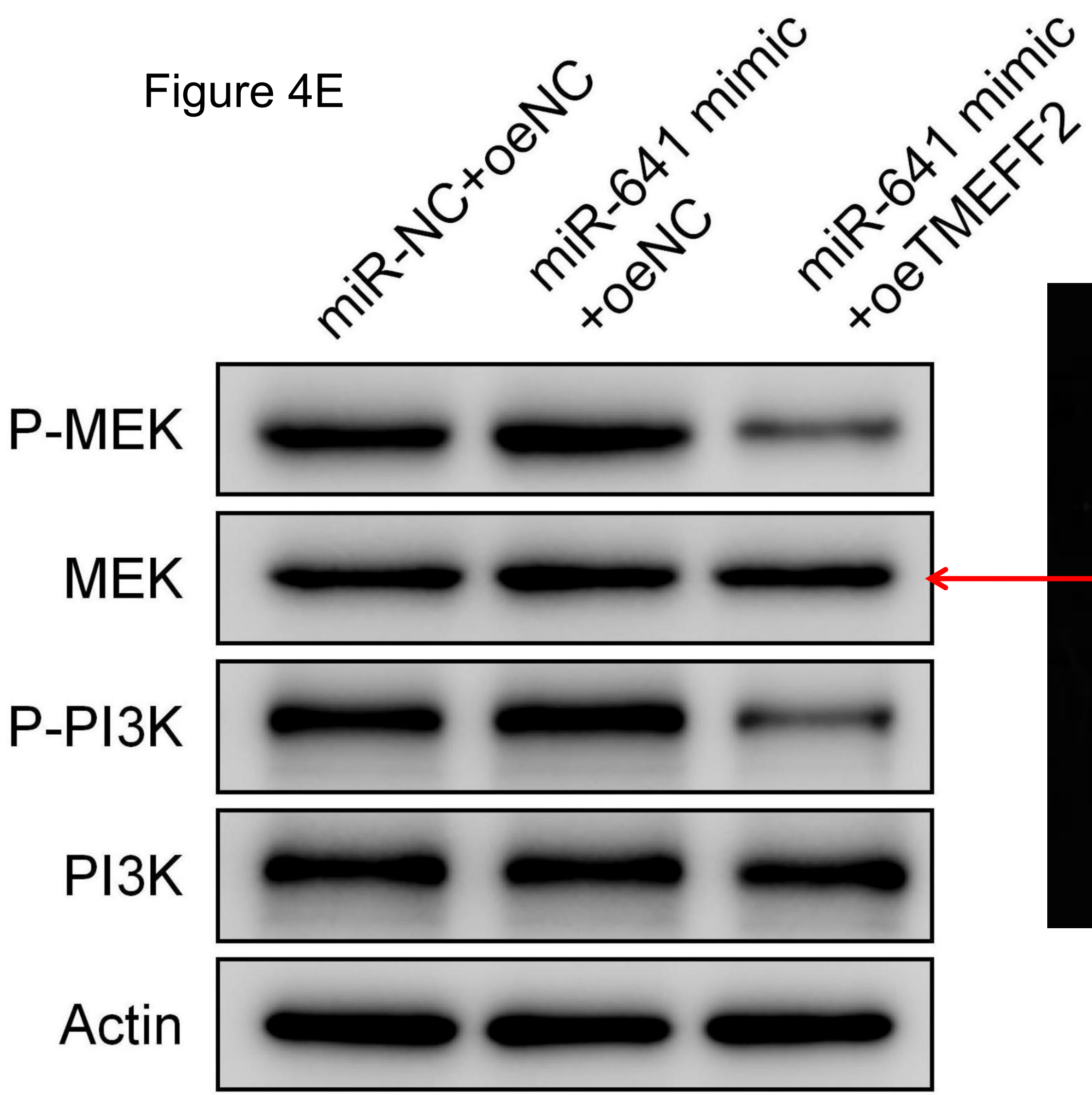

MEK
79kDa

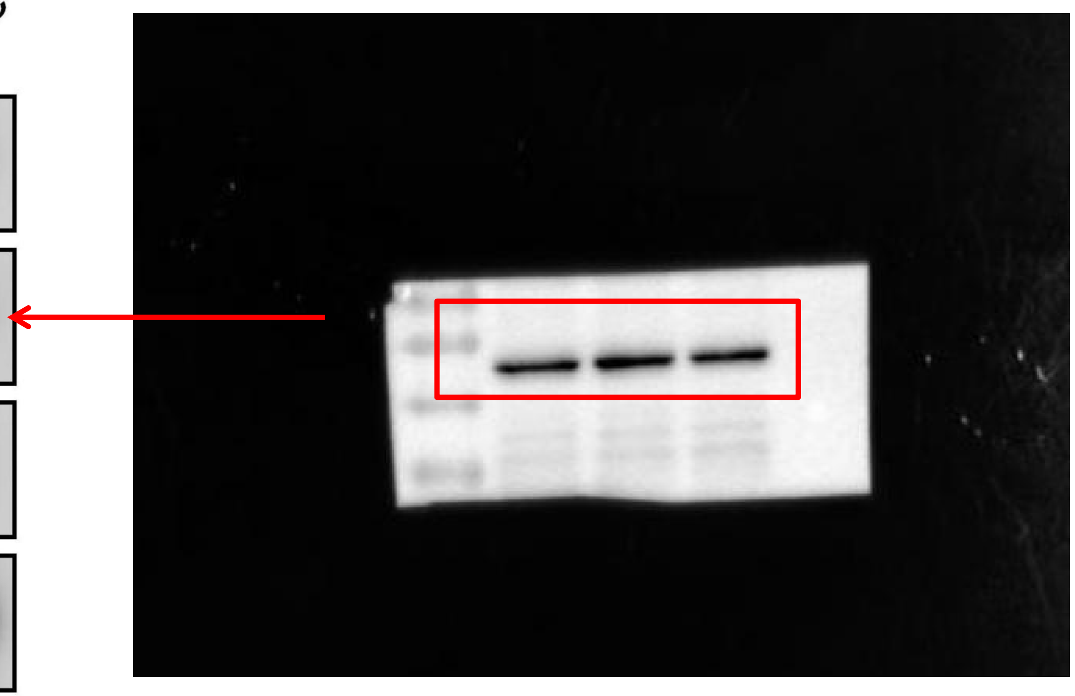

Figure 4E

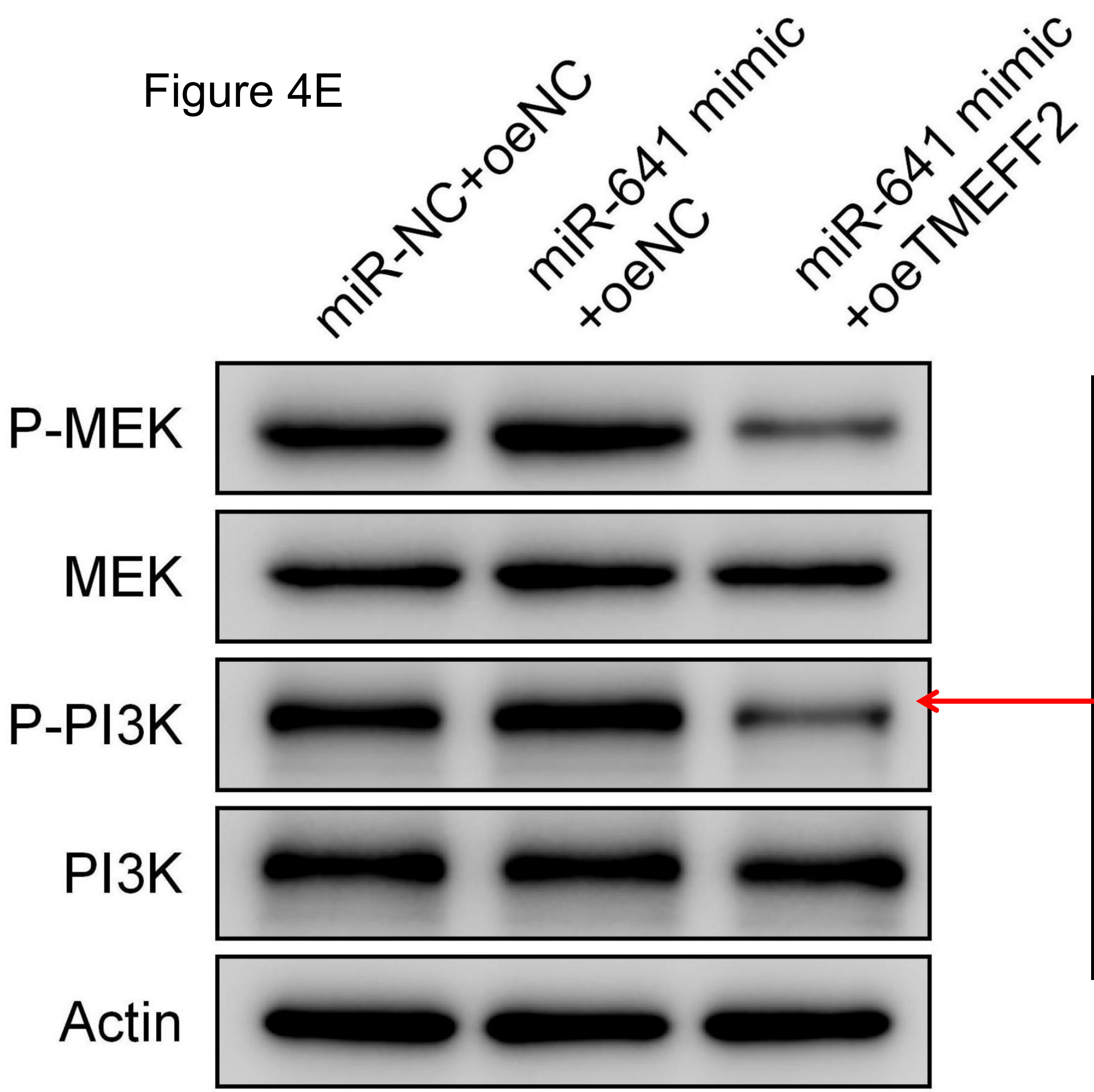

P-PIK3
84kDa

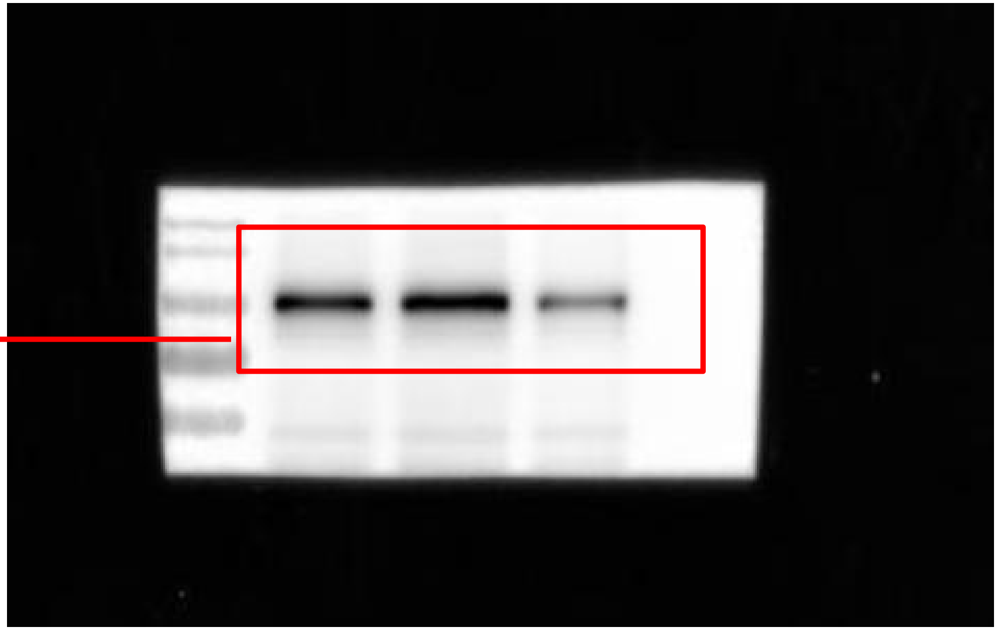

Figure 4E

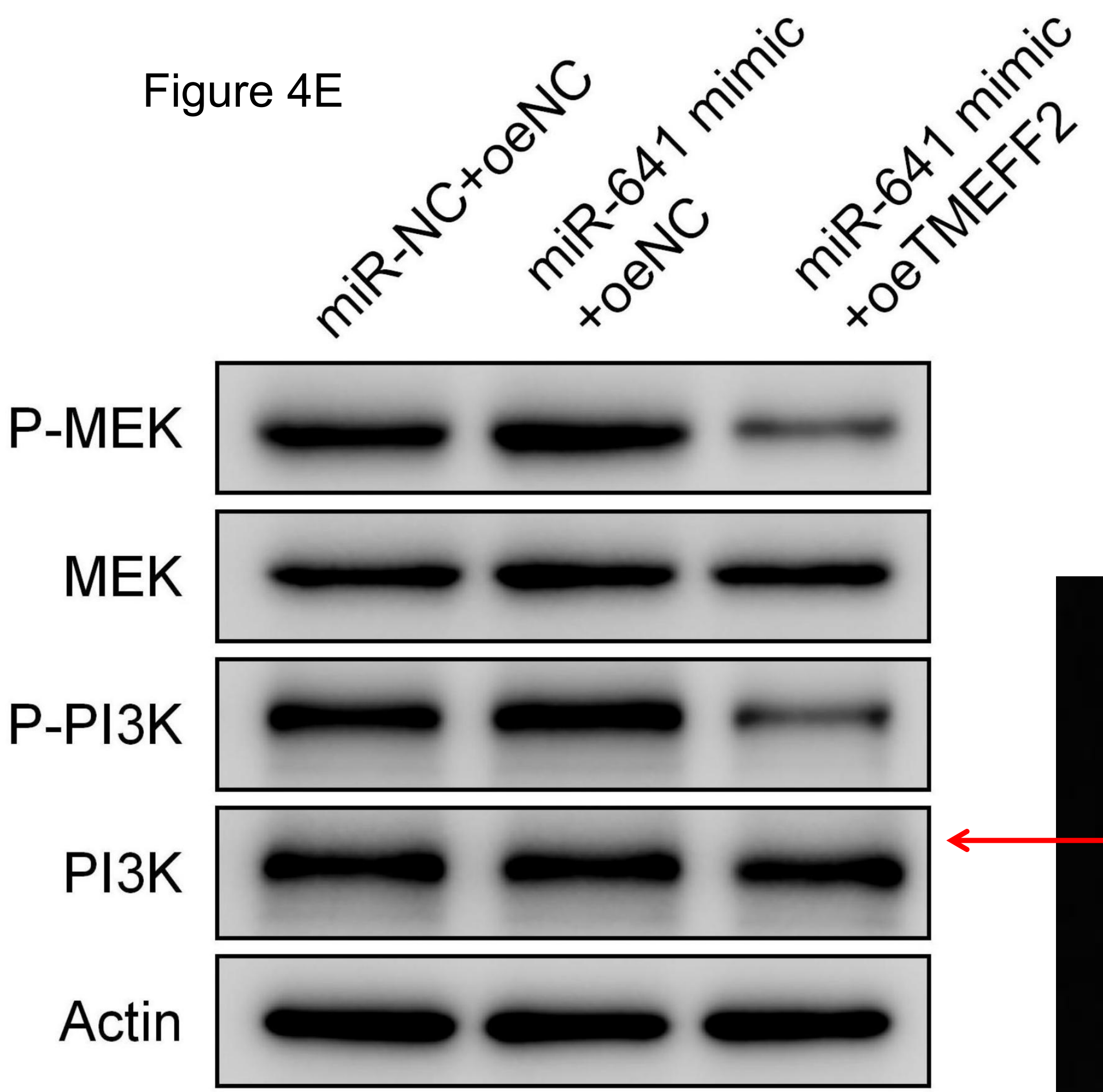

PIK3
126kDa

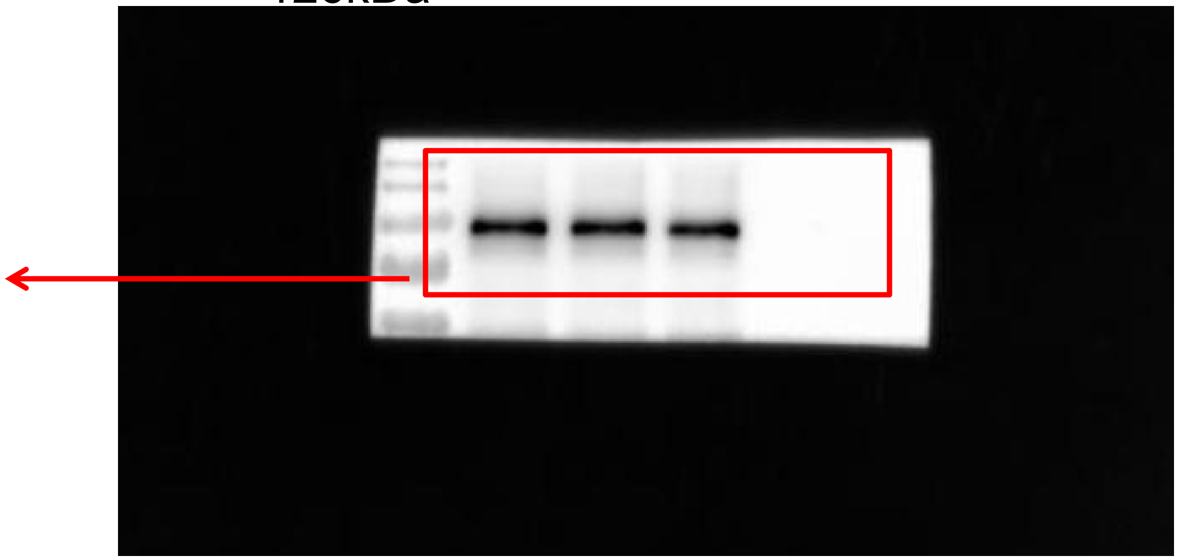

Figure 4E

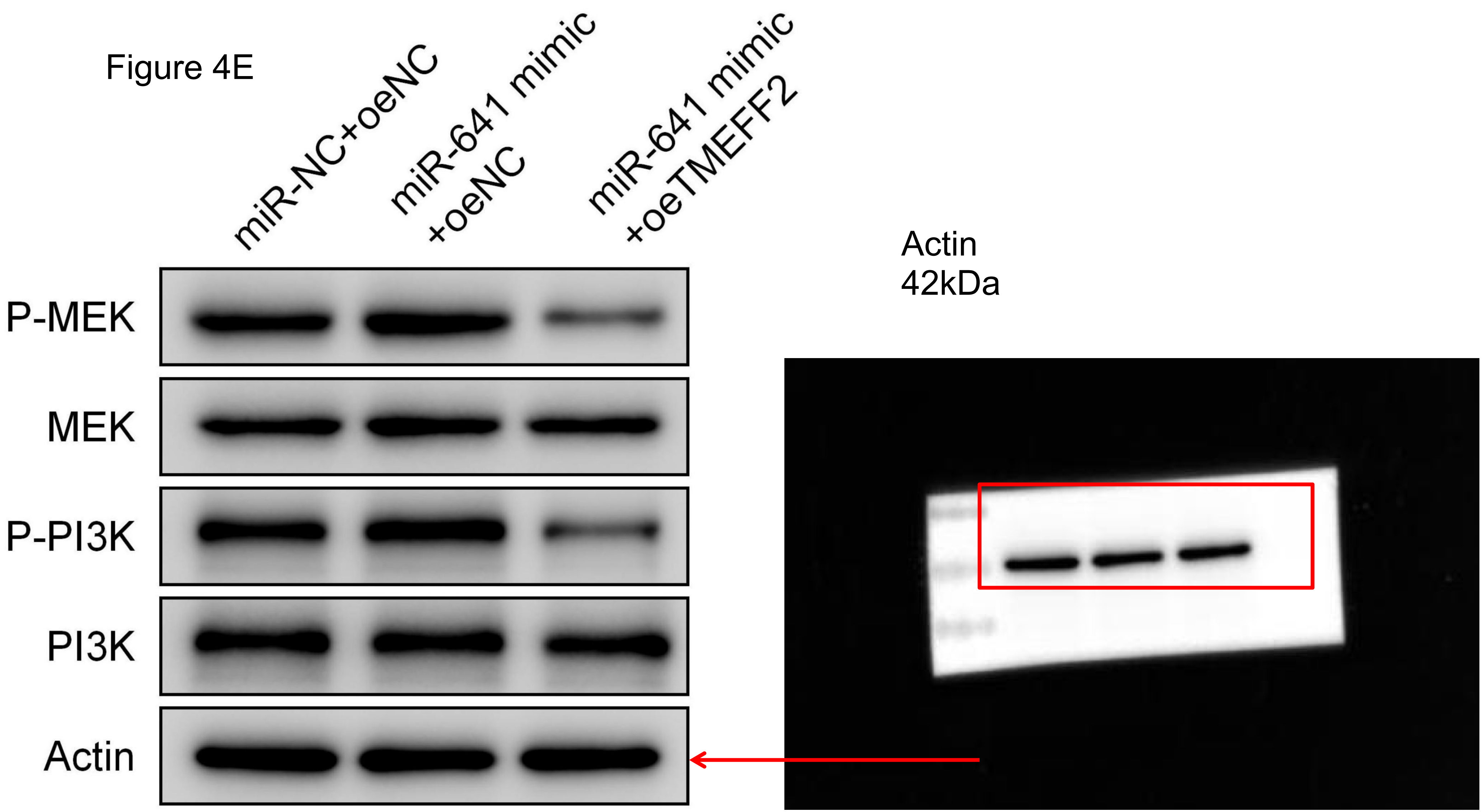

miR-NC+oeNC
miR-641 mimic
+oeNC
miR-641 mimic
+oeTMEFF2
P-MEK
MEK
P-PI3K
PI3K
Actin
Actin
42kDa

Figure 4D

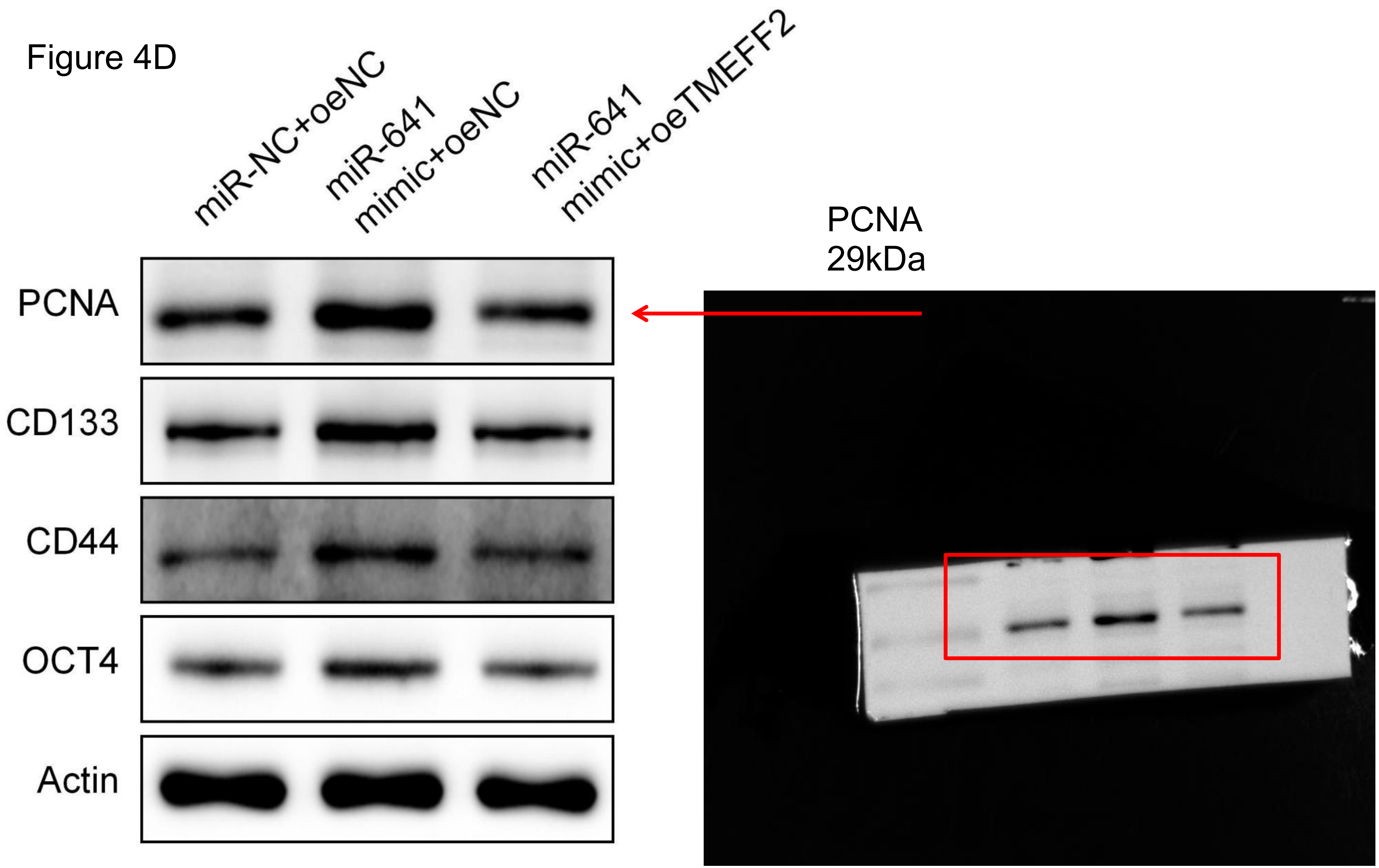
miR-NC+oeNC
miR-641 mimic+oeNC
miR-641 mimic+oeTMEFF2
PCNA
CD133
CD44
OCT4
Actin
PCNA
29kDa

Figure 4D

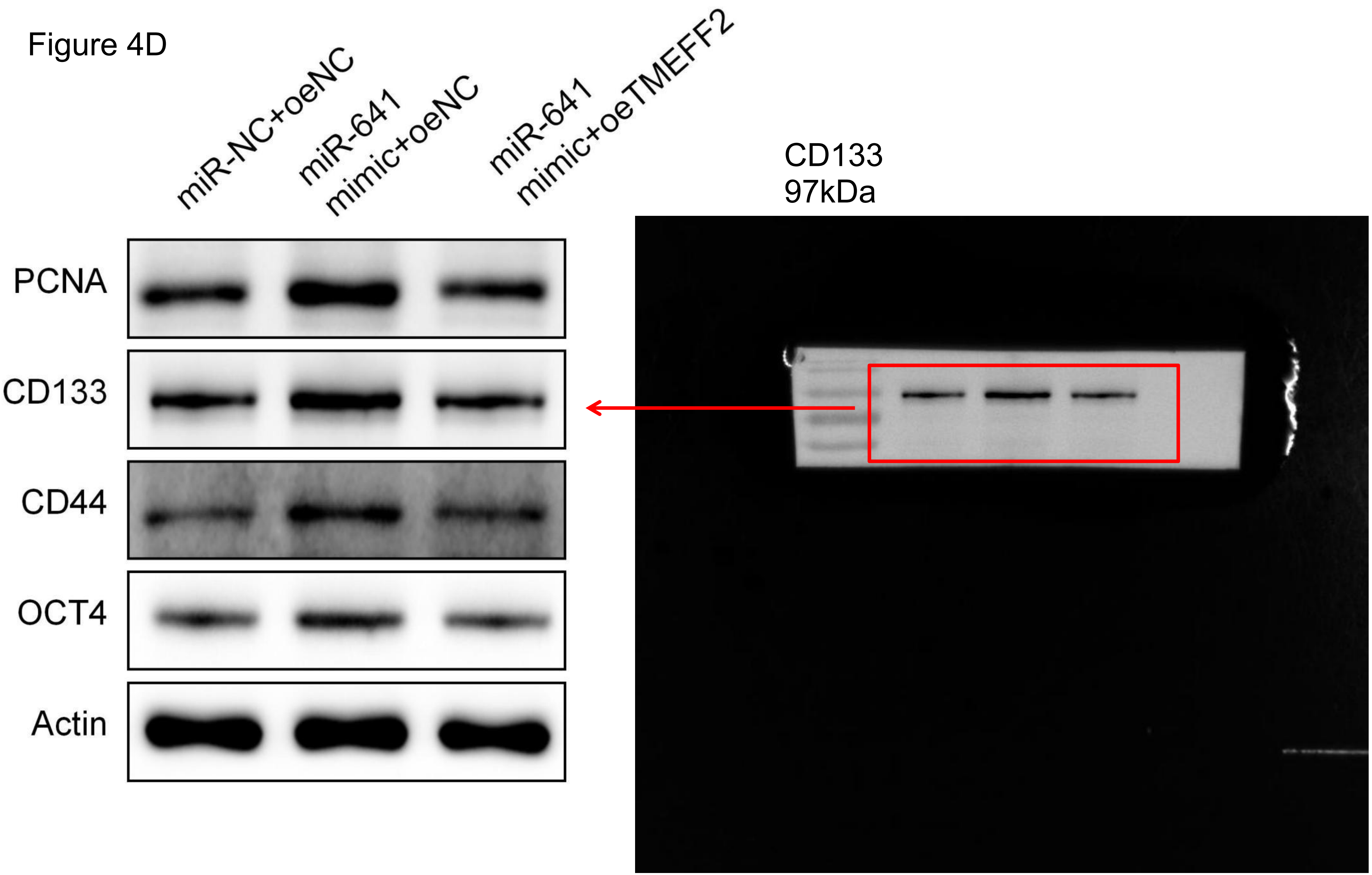

miR-NC+oeNC
miR-641 mimic+oeNC
miR-641 mimic+oeTMEFF2
PCNA
CD133
CD44
OCT4
Actin
CD133
97kDa

Figure 4D

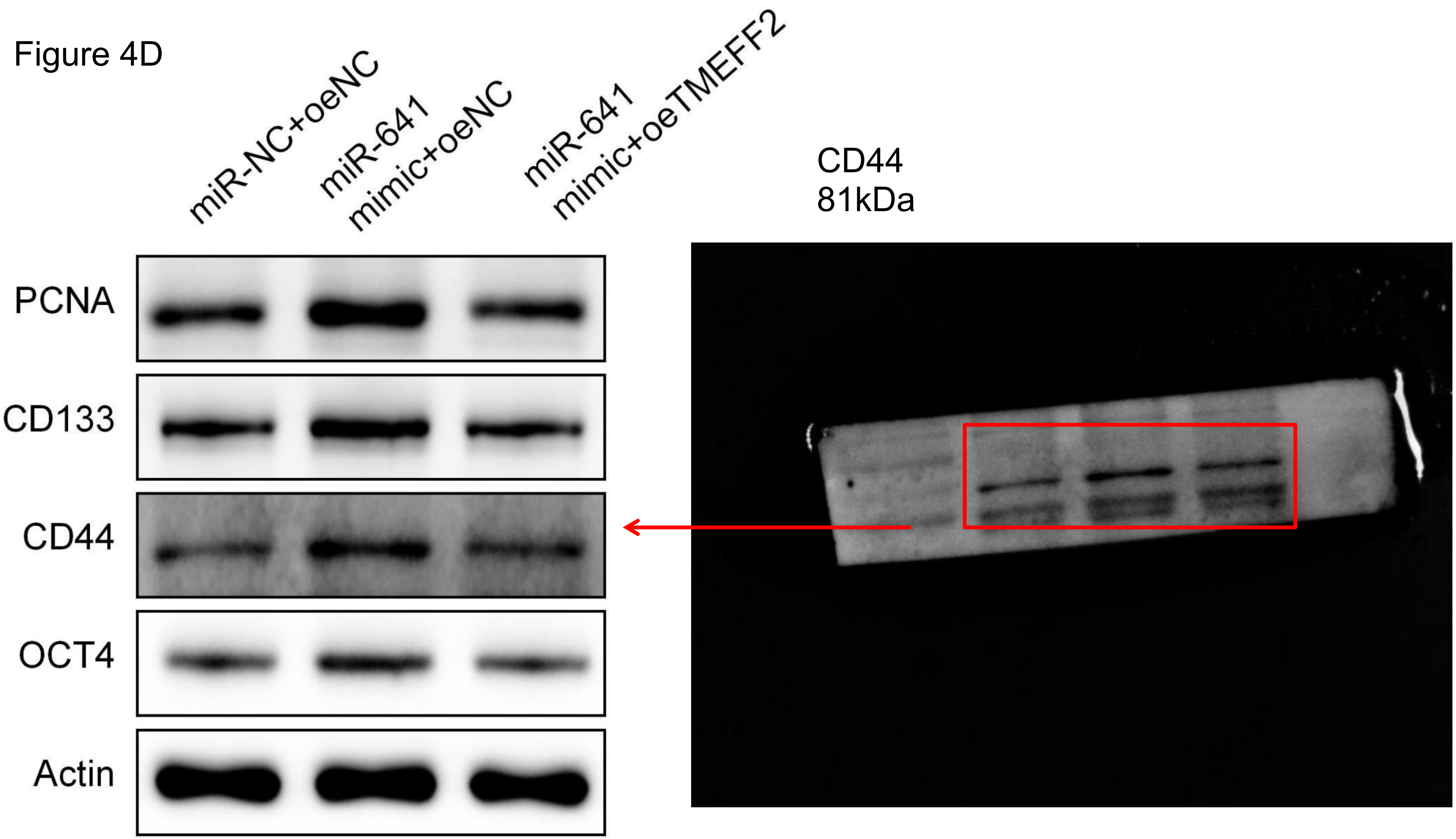

miR-NC+oeNC
miR-641 mimic+oeNC
miR-641 mimic+oeTMEFF2
PCNA
CD133
CD44
OCT4
Actin
CD44
81kDa

Figure 4D

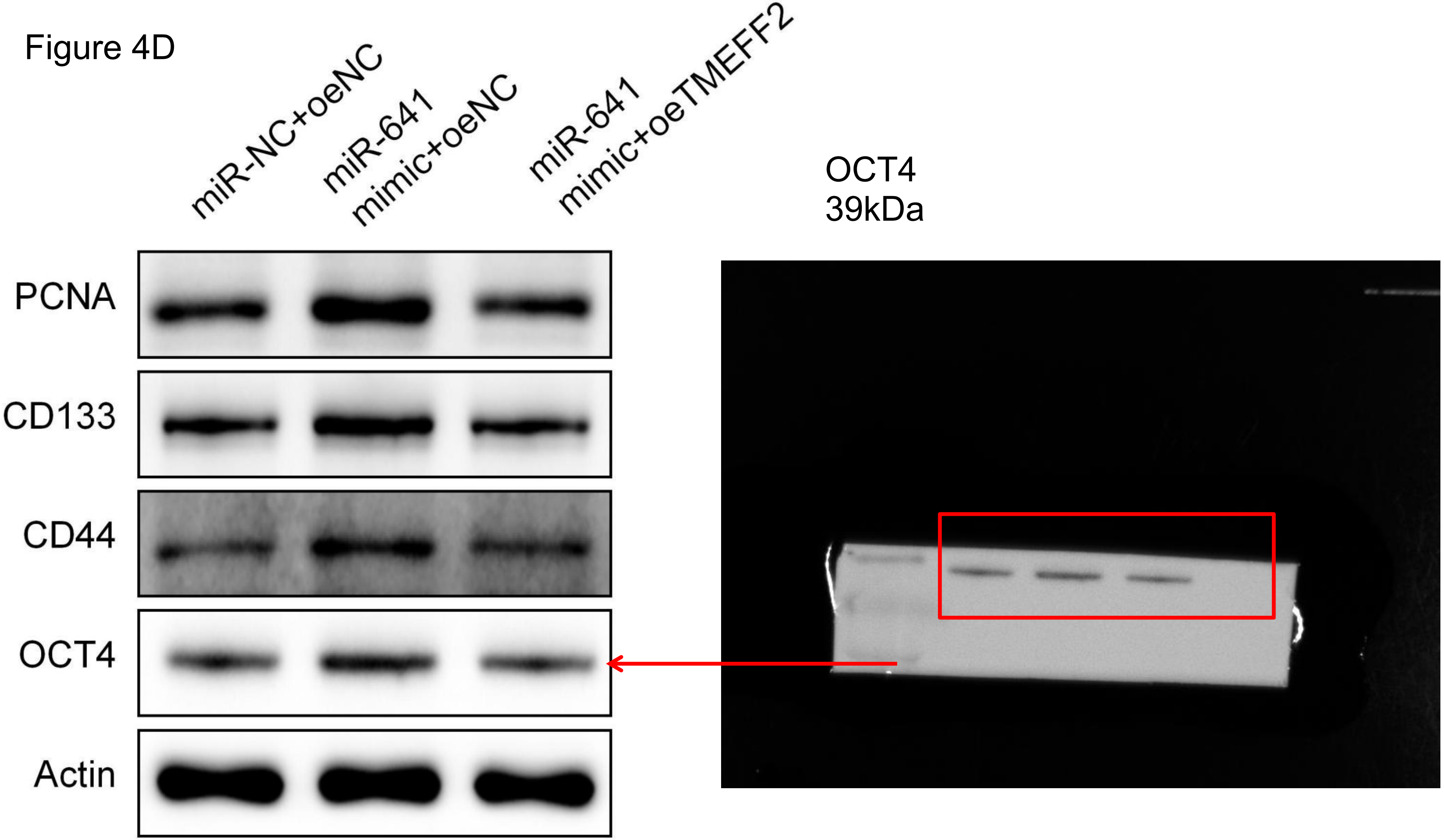
miR-NC+oeNC
miR-641 mimic+oeNC
miR-641 mimic+oeTMEFF2
PCNA
CD133
CD44
OCT4
Actin
OCT4
39kDa

Figure 4D

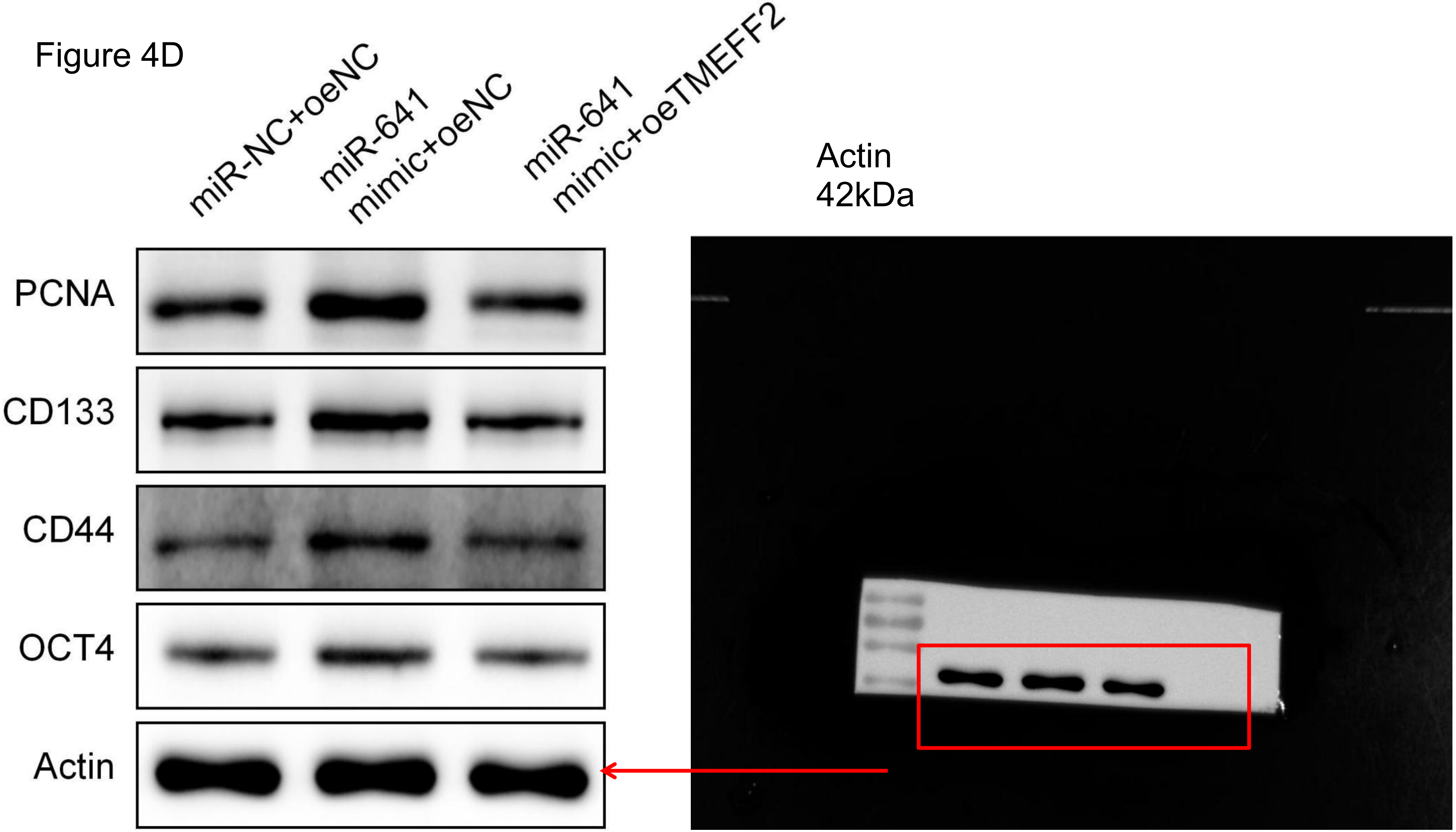
miR-NC+oeNC
miR-641 mimic+oeNC
miR-641 mimic+oeTMEFF2
PCNA
CD133
CD44
OCT4
Actin
Actin
42kDa
